# The Small Molecule Inhibitor QLT-0267 Decreases the Production of Fibrin-Induced Inflammatory Cytokines and Prevents Post-Surgical Peritoneal Adhesions

**DOI:** 10.1038/s41598-018-25994-5

**Published:** 2018-06-21

**Authors:** Cheng-Chung Fang, Tzung-Hsin Chou, Jenq-Wen Huang, Chien-Chang Lee, Shyr-Chyr Chen

**Affiliations:** 1 Departments of Emergency Medicine, National Taiwan University Hospital and National Taiwan University College of Medicine, Taipei, Taiwan; 2 Departments of Internal Medicine, National Taiwan University Hospital and National Taiwan University College of Medicine, Taipei, Taiwan; 3 Departments of Surgery, National Taiwan University Hospital and National Taiwan University College of Medicine, Taipei, Taiwan

## Abstract

Peritoneal adhesions develop after abdominal surgery, trauma or intraperitoneal infections, and have important consequences. The deposition of peritoneal fibrin is a common pathophysiological pathway for the formation of adhesions. Here, we aimed to examine the effects of fibrin-induced cytokine production on peritoneal mesothelial cells (PMCs), and to block the effects of fibrin using an integrin-linked kinase (ILK) inhibitor, QLT-0267. PMCs were cultured from the enzymatic disaggregation of rat omentum. After the PMCs were covered with fibrin, the expression of IL-1β, IL-6, TNFα and VEGF-A increased. This increase in cytokine production was attenuated by QLT-0267, which acted via the inhibition of both the ILK and focal adhesion kinase (FAK) pathways, and subsequently via the GSK-3β pathway. We found that QLT-0267 decreased both the severity of peritoneal adhesion and the serum levels of IL-6 in our post-surgical adhesion mouse model. In conclusion, our study provides novel evidence that fibrin-induced cytokine production may involve in the mechanism of peritoneal adhesion formation. Furthermore, the use of the small molecule inhibitor QLT-0267 is a new strategy in preventing peritoneal adhesion in patients undergoing abdominal surgery.

## Introduction

Intra-abdominal adhesions develop after abdominal surgery, trauma or intraperitoneal infections. Adhesions are the main cause of intestinal obstruction in the developed world^1^, and are reported to cause 32% of acute intestinal obstruction and 65–75% of all small bowel obstructions^2^. Peritoneal adhesions have important consequences to patients, surgeons and the health system^3,4^. Over the decades, there has been much research into the biochemical and cellular processes that lead to adhesion formation and preventions for peritoneal adhesion^5^.

The intra-abdominal formation of fibrin is a common pathophysiological pathway involved in the formation of adhesions^6^. The abdominal cavity is lined by the peritoneum, which consists of a single layer of mesothelial cells and a submesothelial layer. Peritoneal trauma results in mesothelial damage and is accompanied by inflammation. Mesothelial cells detach from the basal membrane and create denuded areas, which results in inflammatory reactions^7^. The inflammatory reaction causes influx of inflammatory cells and leads to a fibrinous exudate^8^. When two peritoneal surfaces coated with fibrinous matrix come into apposition, a band or bridge often forms, which becomes the base around which an adhesion is organised. Regenerating mesothelial cells possess vital peritoneal fibrinolytic activity, and prevent the formation of adhesions via the lysis of fibrin bands^4^. It has been shown that adhesion formation is inversely correlated with the fibrinolytic activity of the peritoneum, and bacterial peritonitis causes fibrinolytic activity to decrease^9,10^. If the fibrinolytic mechanism fails, the adhesions will become fibrous and organized. Therefore, early balance between fibrin deposition and degradation seems to be the critical factor in adhesion formation. Furthermore, the peritoneal inflammatory status appears to be a crucial factor in determining the duration and extent of the imbalance between fibrin formation and dissolution^11^. Suppression of inflammation and augmentation of fibrinolytic activity may be promising anti-adhesion treatment strategies^11^.

Fibrin has been investigated as a matrix to promote wound healing, as demonstrated by several cells interacting with the fibrin matrix. Endothelial cells, smooth muscle cells, fibroblasts and leukocytes can bind directly to fibrin and/or fibrinogen via cell surface integrin receptors and non-integrin receptors^12^. These cells may infiltrate into the fibrin matrix and induce many actions such as angiogenesis^12^, fibroblast proliferation and wound healing with fibrosis^13^. However, the effects of fibrin and fibrinogen in relation to cytokine production in the pathogenesis of peritoneal adhesion have not been elucidated. Therefore, we proposed that fibrin may have proinflammatory effects that contribute to the formation of fibrosis bands, in addition to simply apposing two peritoneal organs to initiate adhesion.

Various strategies have been employed over many years to prevent adhesion formation, such as reducing peritoneal damage by using laparoscopic surgery; preventing fibrin formation using heparin; inhibiting inflammatory reactions using steroids, non-steroidal anti-inflammatory drugs or vitamin E; promoting fibrinolysis using thrombolytic agents; and the use of physical barriers^14–16^. At present, minimizing peritoneal damage during surgery is the most important strategy to prevent adhesion^2,14,17^. Recently, a meta-analysis that included 28 trials (5191 patients) concluded that oxidised regenerated cellulose and hyaluronate carboxymethylcellulose can reduce adhesions^18^. However, there still are concerns that the use of barriers may increase the incidence of anastomotic leakage^2^. As our understanding of the specific mechanisms involved in peritoneal repair evolves, it seems likely that specific targets for adhesion prevention could be employed^1^. Many animal studies concluded that the postoperative intraperitoneal administration of thrombolytic agents, typically recombinant tissue plasminogen activator (rtPA), can significantly decrease adhesion formation^19–21^. However, the use of rtPA is limited due to the requirement for intraperitoneal administration and the risk of haemorrhage^1,22^. A prospective randomized clinical study showed that rtPA did not decrease post-surgical adhesion, probably due to inadequate rtPA administrated^23^. Therefore, the clinical use of rtPA in preventing adhesion is challenged by its ineffectiveness and risk of bleeding complications^14,24^.

In this study, we aimed to examine the effects of fibrin-induced cytokine production on peritoneal mesothelial cells (PMCs). We proposed that fibrin could induce cytokine production from PMCs, which augments peritoneal inflammation and leads to peritoneal adhesions. We also attempted to manipulate the effects of fibrin at the cellular level via its signalling transduction target; specifically, we aimed to block fibrin-induced effects by inhibiting the integrin-linked kinase (ILK) pathway. Recently, an ILK inhibitor, QLT-0267, has been investigated in cancer treatment^25–27^ and interstitial renal fibrosis attenuation^28^. Therefore, we aimed to study the *in vitro* and *in vivo* effects of QLT-0267 on peritoneal adhesion.

## Methods

### Ethics Statement

All rat and mouse experiments were performed according to the regulations of the American Association for Accreditation for Laboratory Animal Care, and were approved by the Institutional Animal Care and the Use Committee of National Taiwan University College of Medicine (Approval #20110536).

### Materials

QLT-0267 was kindly provided by Valocor Therapeutics, Inc. (Vancouver, Canada). All chemicals were obtained from Sigma-Aldrich (St. Louis, MO, USA) unless otherwise specified. The immunoassay kits for IL-1β, IL-6, VEGF-A, TNFα and TGFβ were purchased from R&D Systems (Minneapolis, MN, USA). TRIzol® reagent was purchased from Life Technologies (Carlsbad, CA, USA). RevertAid H Minus Reverse Transcriptase® was purchased from Thermo Fisher Scientific (Waltham, MA, USA). Taq DNA Polymerase Master Mix Red® was purchased from Ampliqon (Odense M, Denmark).

### Peritoneal Mesothelial Cell Culture

Omentum specimens were obtained from sacrificed rats. The method for enzymatic disaggregation of the omentum was performed as previously described^29^. The rat PMCs initially appeared bipolar or multipolar, but became cobblestone-like in appearance upon confluence. The PMCs were identified by the presence of cytokeratin-18, and the absence of α-smooth muscle actin (Supplementary Figure 1S). PMCs were cultured to sub-confluence on 6-well plates in Roswell Park Memorial Institute (RPMI) medium supplemented with 10% foetal bovine serum (FBS). Fibrinogen (10 mg/ml in RPMI) with 0.2 U/ml thrombin was added on top of the cells to form fibrin clots. In experiments involving QLT-0267, agents were pre-treated for 30 min, added to the fibrinogen solution, and then added to the covered RPMI medium at various concentrations. At varying time intervals, the clots were removed, and the cells were harvested for protein or mRNA extraction or immunofluorescent staining.

### Reverse Transcription PCR (RT-PCR)

Total RNA was extracted using the TRIzol RNA isolation system (Invitrogen, Carlsbad, CA, USA), and purity was determined by A260/A280 values. First-strand cDNA synthesis was performed by reverse transcription, using 2 μg of total RNA in 20 μl of reaction buffer with RevertAid™ H Minus Reverse Transcriptase (Thermo Fisher Scientific, Waltham, MA, USA). PCR amplification of cDNA was carried out using a standard PCR kit and 1 μl of cDNA. The specific primers used for each gene are listed in Supplementary Table S1. PCR products were electrophoresed on 1% agarose gels and stained with DNA View (TOOLS, Taipei, Taiwan) to visualize DNA bands. Images were captured (BioDoc-It® Imaging System) and quantitated using GelPro analysis software.

### Quantitative Real-Time PCR (qPCR)

After RNA extraction, first-strand cDNA synthesis was performed using the iScript cDNA synthesis kit (Bio-Rad, Hercules, CA, USA) with 1 μg of RNA in 20 μl of reaction buffer. qPCRs were performed in 20 μl reaction mixtures containing 10 μl of iQ™ SYBR Green Supermix (Bio-Rad), 0.5 μM of each forward and reverse primer and 1 μl of cDNA template. Reactions were performed in the Bio-Rad MyiQ™ single colour real-time PCR detection system using the following cycling parameters: 5 min at 95 °C, followed by 40 cycles of 10 s at 95 °C and 30 s at 60 °C. The specific primers used for each gene are listed in Supplementary Table S2. Gene expression was normalized to 18S rRNA, a housekeeping gene, and expressed as fold change relative to control, calculated with the ΔΔCT method.

### Western Blotting

PMCs were lysed in ice-cold radioimmunoprecipitation (RIPA) buffer (50 mM Tris, pH 8.0, 150 mM NaCl, 1% IGEPAL, 0.5% Na deoxycholate, 0.1% SDS) with protease and phosphatase inhibitors. Samples were rotated for 15 min at 4 °C and then centrifuged at 16,000 *g* for 15 min at 4 °C. The resulting supernatant was recovered, and protein concentrations were measured using a Bradford Protein Assay kit (Bio-Rad) using bovine serum albumin (BSA) as the standard. Samples were incubated for 5 min at 95 °C in loading buffer (62.5 mM Tris-HCl, pH 6.8, 10% glycerol, 2% SDS, 5% 2-mercaptoethanol, 0.002% bromophenol blue), and 20 μg of protein was loaded on 10% SDS-polyacrylamide gels. After electrophoresis, the proteins were transferred to a polyvinylidene difluoride (PVDF) membrane (Millipore, Bedford, MA, USA). The membrane was blocked with 5% BSA for 1 hour at room temperature, and probed with primary antibodies at 4 °C overnight. The primary antibodies used were as follows: anti-ILK (3856; Cell Signaling Technology, Beverly, MA, USA), anti-Akt (Cell Signaling Technology, 9272), anti-Phospho-Akt (Ser473; Cell Signaling Technology, 9271), anti-GSK-3β (Cell Signaling Technology, 9315), anti-Phospho-GSK-3β (Ser9; Cell Signaling Technology, 9336), anti-NF-κB p65 (sc-8008; Santa Cruz Biotechnology, Dallas, TX, USA), anti-Phospho-NF-κB p65 (Ser536; Cell Signaling Technology, 3033), anti-IκBα (Cell Signaling Technology, 4814), anti-focal adhesion kinase (FAK; Cell Signaling Technology, 3285), anti-Phospho-FAK (Tyr397; Cell Signaling Technology, 3283), and anti-β-actin (Sigma-Aldrich, A5316). Horseradish peroxidase-conjugated anti-mouse or anti-rabbit IgG was used as secondary antibodies. Blots were developed by incubation with a chemiluminescence substrate, and were captured using a BioSpectrum® imaging system (UVP, Inc., LCC, Upland, CA, USA). Images were quantitated using GelPro analysis software.

### Lentiviral-Mediated Knockdown of ILK in PMCs

A lentivirus with ILK short hairpin RNA (shRNA) and a lentivirus with an empty vector were purchased from the National RNAi Core Facility of Academia Sinica (Taipei, Taiwan). The shRNA target sequence of the rat ILK was 5′-TCAGAGCTTTGTCACTTGCCA-3′ (TRCN0000000968), and the empty shRNA vector (pLKO.1-emptyT; TRCN208001) was used as a control. PMCs were plated overnight and then infected with the lentiviruses at a multiplicity of infection (MOI) of 3 in the presence of polybrene (8 μg/ml) for 24 hours. Infected cells were selected using puromycin (4 μg/ml) for three days. The knockdown efficiency of ILK was confirmed by qPCR with ILK-specific primers, or Western blotting with the anti-ILK antibody.

### Animal Model of Post-Surgical Adhesion

The animal model of post-surgical adhesion used was modified as previously described^30^. All experiments were conducted using a protocol approved by our Institutional Animal Care and Use Committee. Adult male ICR mice weighing approximately 30 g were inducted in an anaesthetic chamber with 3% isoflurane, and then maintained at 1.2% isoflurane in medical air through a dedicated nose cone under spontaneous breathing conditions. Isoflurane concentration was regulated with a vaporizer (Bickford Vapomatic, AM Bickford Inc., Wales Center, NY, USA). Briefly, the abdominal cavity was exposed via a midline incision. Using a gauze swab, a 2 cm^2^ section of the parietal peritoneum was removed from each side of the abdominal wall. The treatment by gauze induced fibrin depositions on the injured parietal peritoneum (Supplementary Figure 2S). After administration of the appropriate treatments described below, the incision was closed with sutures, and the mice were returned to their cages for recovery. For preventing postoperative pain, buprenorphine (0.05 mg/kg s.c, twice daily) was administered during the two postoperative days. Mice were randomized to one of three groups: Group 1 received 0.2 ml of saline in the abdominal cavity immediately after a sham operation (i.e., only midline incision); Group 2 received 0.2 ml saline in the abdominal cavity immediately after the operation; and Group 3 received 10 mg/kg of QLT-0267 in 0.2 ml saline in the abdominal cavity immediately after the operation. The mice were sacrificed to collect blood by cardiac puncture at each time point. One, four and eight hrs blood samples were assessed by ELISAs for TNFα, IL-1β, IL-6, TGFβ1 and VEGF-A. The adhesion scores were counted according to the adhesion scoring system (Supplementary Table S3) one or seven post operation day(s). The examples of gross appearance in abdominal cavity are shown in Supplementary Figure 3S. The mice tissue section photos are displayed in Supplementary Figure 2S. The sum of the adhesion number scores and area scores were compared among the groups.

### Statistical Analysis

All data were expressed as mean ± standard error of the mean (SEM) from three independent experiments, unless otherwise indicated in the figure legends. RT-PCR data were compared with one-way ANOVAs followed by Tukey’s *post-hoc* analysis. The gross adhesion scores among groups were compared by the non-parametric Kruskal-Wallis test. The non-parametric Mann-Whitney *U* test was used to determine the differences between each pair of groups. *P* values below 0.05 were considered significant.

## Results

### Fibrin induced cytokine expression from PMCs, which was attenuated by QLT-0267

**Figure 1:**
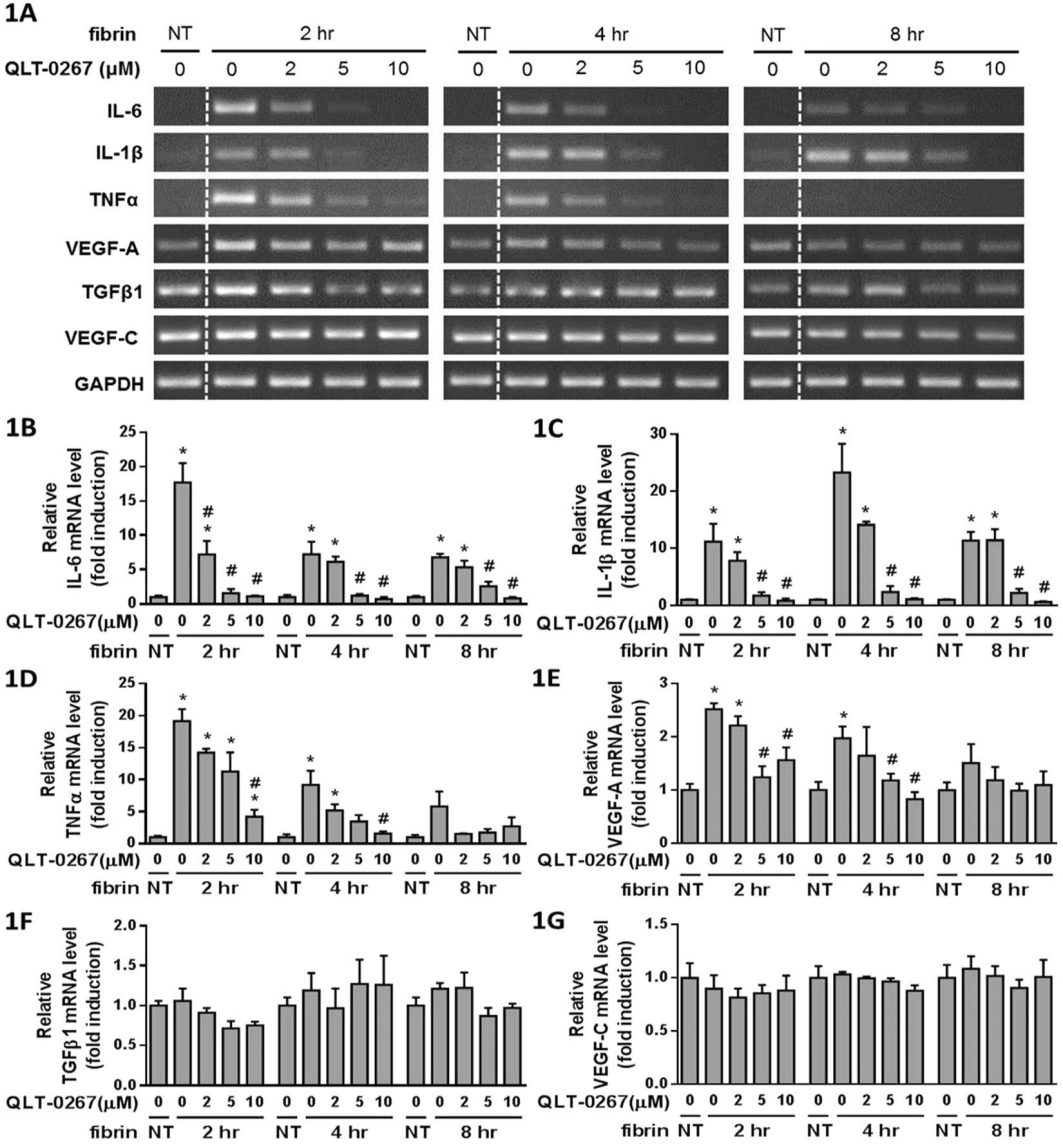
PMCs were overlaid with fibrin via incubation with a fibrinogen and thrombin mixture for 2, 4 and 8 hr. (**A**) The effects of QLT-0267 on cytokine expression were evaluated in triplicate by RT-PCR. The relative expression of (**B**) IL-6, (**C**) IL-1β, (**D**) TNFα, (**E**) VEGF-A, (**F**) TGFβ1 and (**G**) VEGF-C vs. GAPDH are presented as a percentage of increase over untreated PMCs (NT). The line between NT and 0 μM of QLT-0267 was removed (marked by a dotted line), and the original full-length images are presented in Supplementary Figure 4S. *P < 0.05 vs. NT; ^#^*P* < 0.05 vs. 0 μM of OLT-0267 at the same incubation time.

PMC cultures were overlaid with fibrin via incubation with a mixture of fibrinogen and thrombin for two, four and eight hours, and the expression of IL-6, IL-1β, TNFα, VEGF-A, TGFβ1 and VEGF-C were evaluated by RT-PCR (Fig. 1A). Treating PMCs with fibrin led to increased expression of IL-6 (Fig. 1B) and IL-1β (Fig. 1C) at each time interval studied, which were all attenuated by QLT-0267. The expression of TNFα (Fig. 1D) and VEGF-A (Fig. 1E) increased at two and four hours after fibrin treatment, and were both attenuated by QLT-0267. However, the expression of TGFβ1 (Fig. 1F) and VEGF-C (Fig. 1G) were unaffected by fibrin treatment.

### Signal transduction pathways in fibrin-induced cytokine expression were affected by QLT-0267

**Figure 2:**
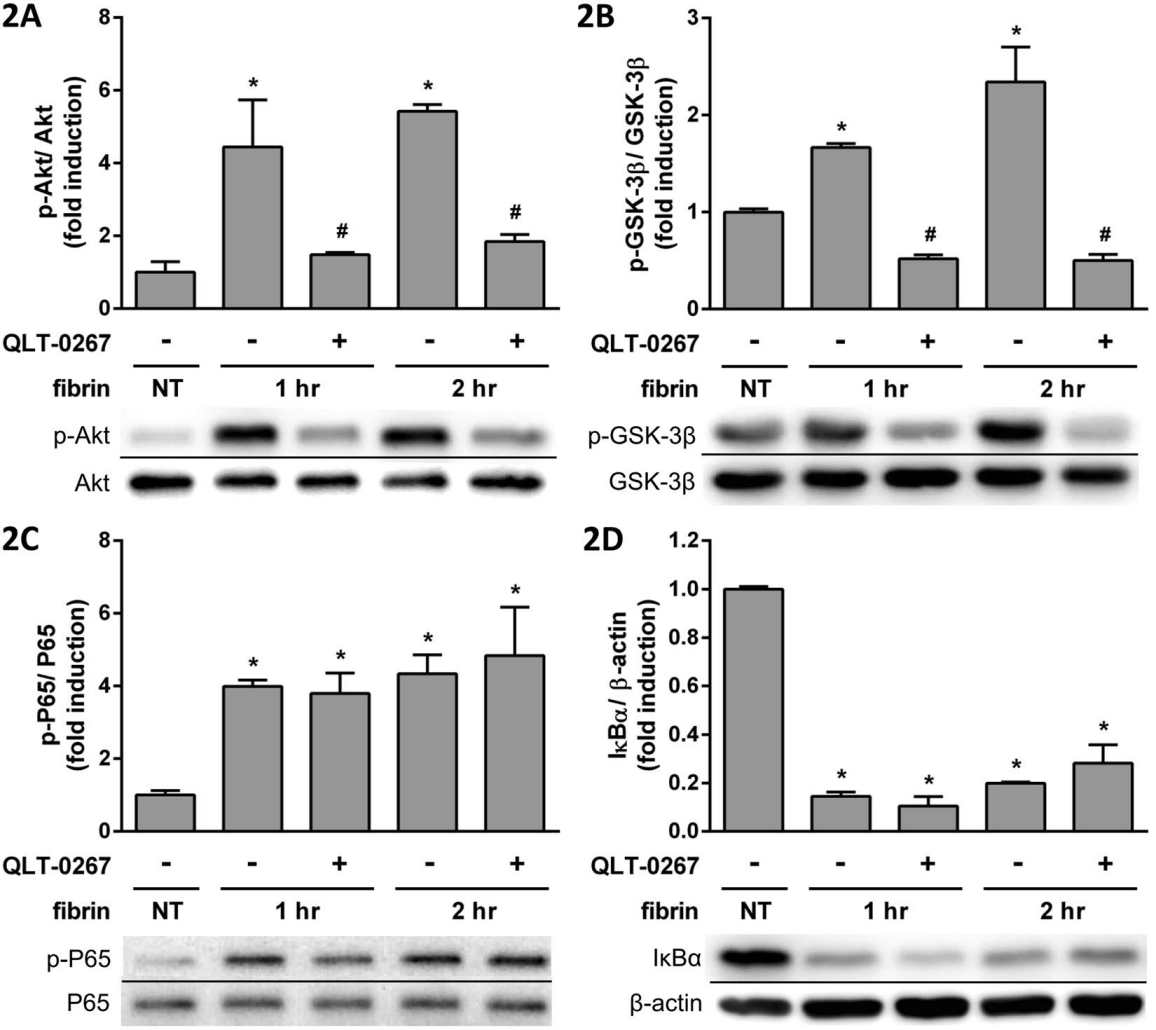
PMCs were overlaid with fibrin via incubation with a fibrinogen and thrombin mixture for 0, 1 and 2 hr. The effects of QLT-0267 treatment on the expression of transduction factors were evaluated in triplicate by Western blotting. The relative expression of (**A**) p-Akt vs. Akt, (**B**) p-GSK-3β vs. GSK-3β, (**C**) p-P65 vs. P65 and (**D**) IκBα vs. β-actin are presented as a percentage of increase over untreated PMCs (NT). *P < 0.05 vs. NT; ^#^*P* < 0.05 vs. fibrin only at the same incubation time.

We examined the Akt, glycogen synthase kinase 3β (GSK-3β), P65 and IκBα signal transduction pathways by Western blots. Phosphorylated Akt (p-Akt) increased after fibrin treatment, and was attenuated by QLT-0267 (Fig. 2A). Phosphorylated Ser9 GSK-3β (p-GSK-3β), which decreases GSK-3β activity, also increased after fibrin treatment, and was attenuated by QLT-0267 (Fig. 2B). Phosphorylated P65 (p-P65) increased after fibrin treatment, and was unaffected by QLT-0267 (Fig. 2C). IκBα decreased after fibrin treatment, and was unaffected by QLT-0267 (Fig. 2D).

**Figure 3:**
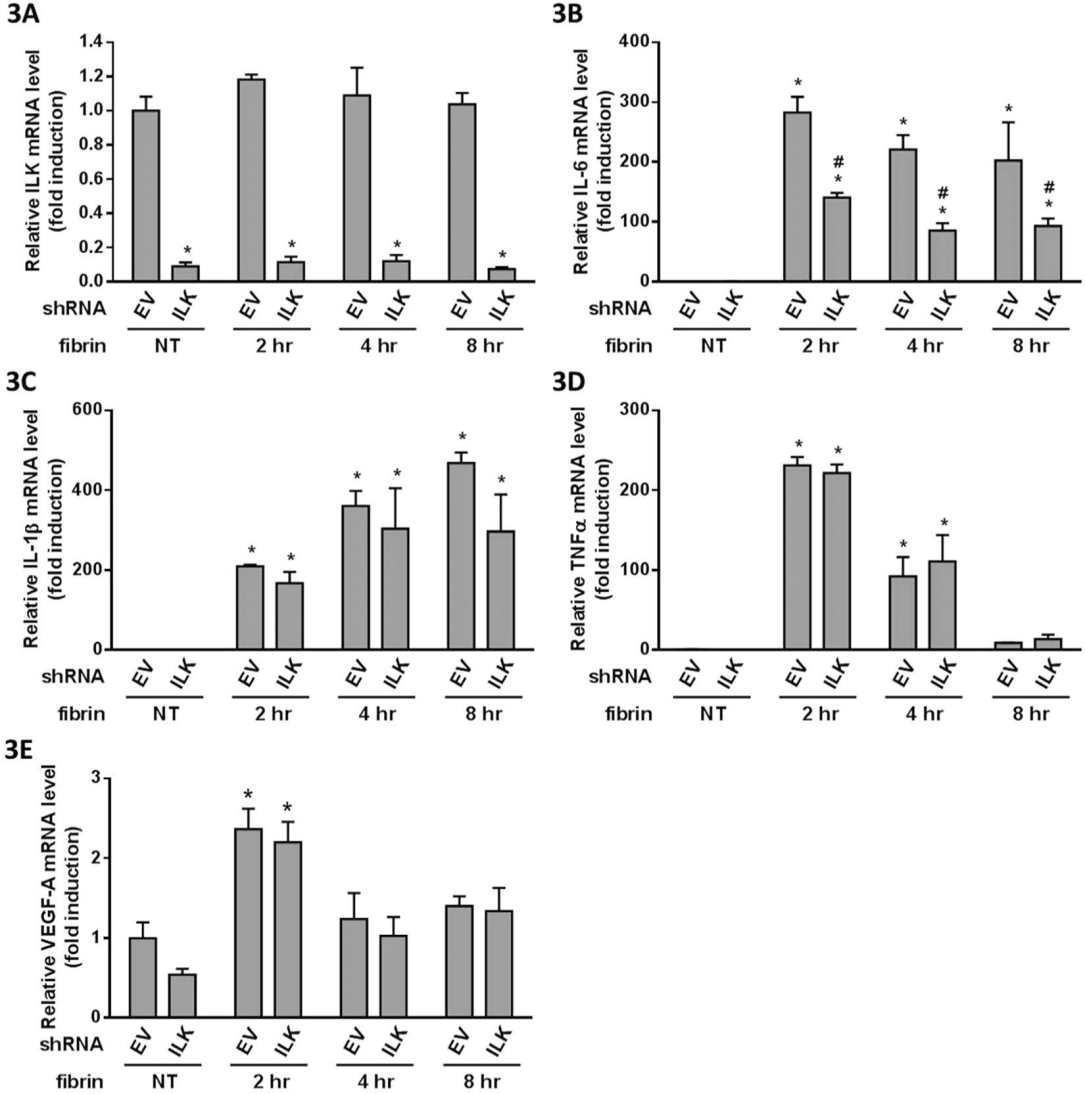
PMCs were infected with lentiviruses carrying either shILK (ILK) or an empty vector (EV) control. PMCs were overlaid with fibrin via incubation with a fibrinogen and thrombin mixture for 0, 2, 4 and 8 hr. Expression of cytokine mRNA was evaluated in triplicate by qPCR. The qPCR data for (**A**) ILK, (**B**) IL-6, (**C**) IL-1β, (**D**) TNFα and (**E**) VEGF-A are presented as a percentage of increase over untreated PMCs (NT). *P < 0.05 vs. NT; ^#^*P* < 0.05 vs. EV at the same incubation time.

**Figure 4:**
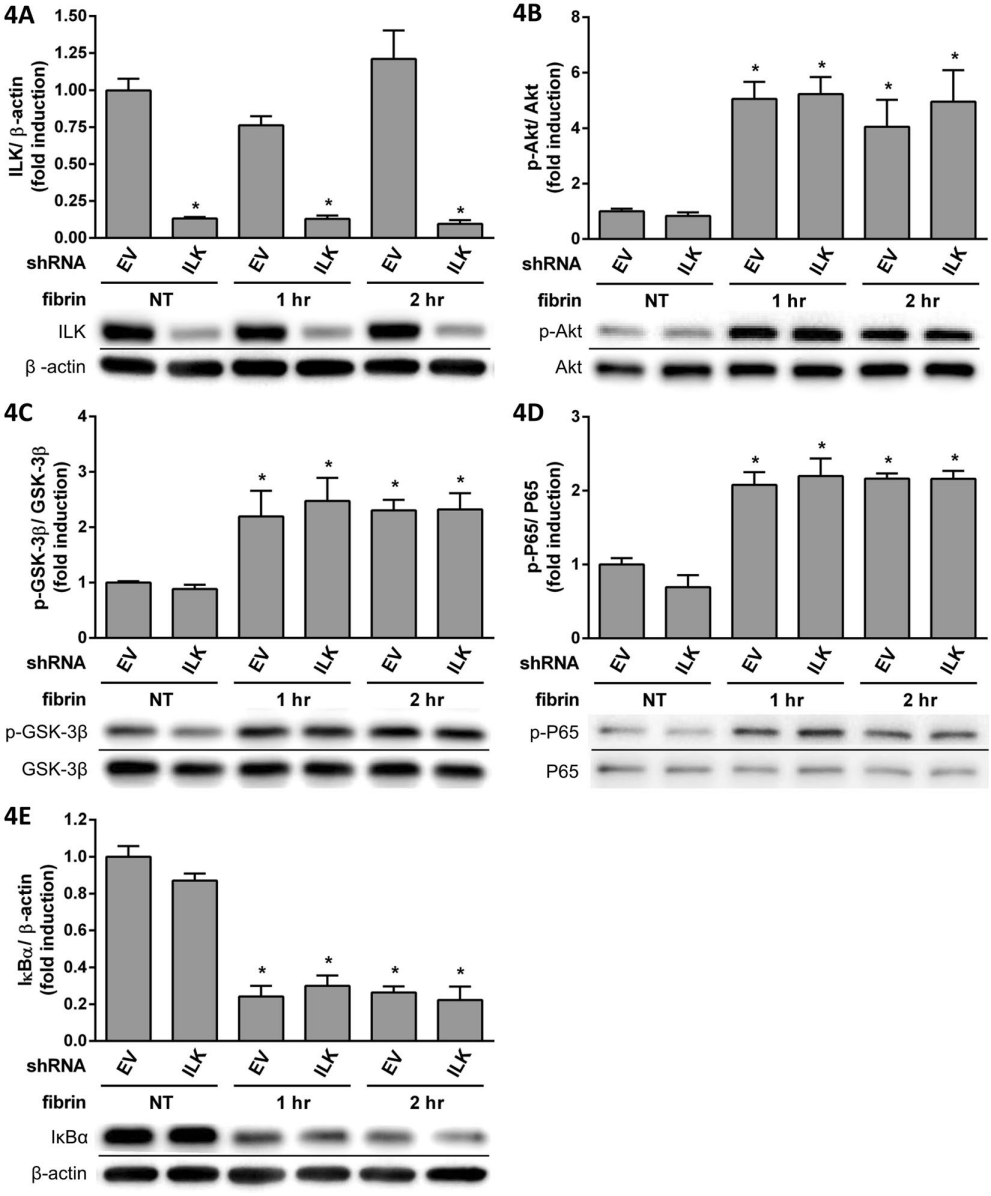
PMCs were infected by lentiviruses carrying either shILK (ILK) or an empty vector (EV) control. PMCs were overlaid with fibrin via incubation with a fibrinogen and thrombin mixture for 0, 1 and 2 hr. Expression of transduction factors were evaluated in triplicate by Western blotting. The relative expression of (**A**) ILK vs. β-actin, (**B**) p-Akt vs. Akt, (**C**) p-GSK-3β vs. GSK-3β, (**D**) p-P65 vs. P65 and (**E**) IκBα vs. β-actin are presented as a percentage of increase over untreated PMCs (NT). *P < 0.05 vs. NT; ^#^*P* < 0.05 vs. EV at the same incubation time.

**Figure 5:**
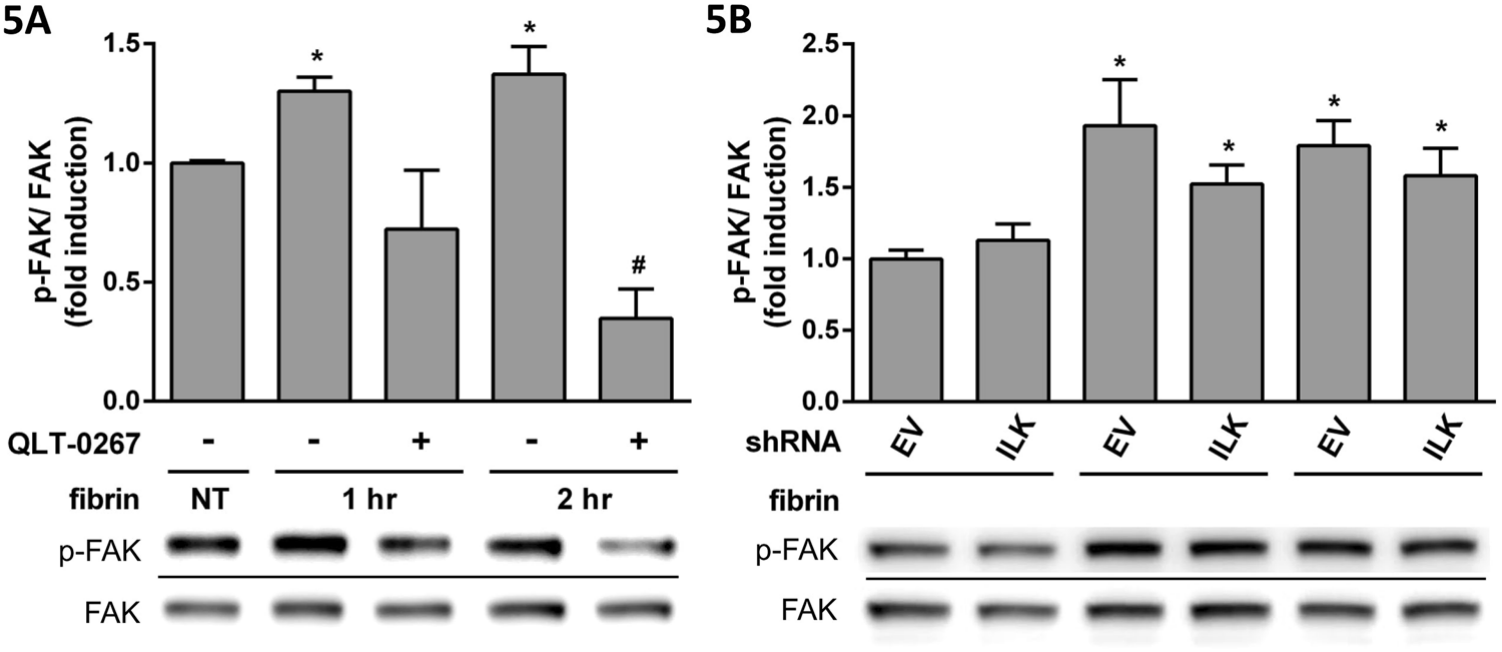
PMCs were overlaid with fibrin via incubation with a fibrinogen and thrombin mixture for 0, 1 and 2 hr. The effect of QLT-0267 treatment on p-FAK expressions was evaluated in triplicate by Western blotting. (**A**) The relative expressions of p-FAK vs. FAK are presented as a percentage of increase over untreated PMCs (NT). PMCs infected with lentiviruses carrying either shILK (ILK) or an empty vector (EV) control were overlaid with fibrin via incubation with a fibrinogen and thrombin mixture for 0, 1 and 2 hr. p-FAK expression was evaluated in triplicate by Western blotting. (**B**) The relative expression of p-FAK vs. FAK is presented as percentage of increase over NT. *P < 0.05 vs. NT; ^#^*P* < 0.05 vs. fibrin only at the same incubation time.

Because QLT-0267 was shown to be an ILK inhibitor^26^, we examined the effects of ILK knockdown by treating PMCs with shILK (Fig. 3A). Fibrin-induced IL-6 expression was suppressed after shILK treatment (Fig. 3B), but IL-1β (Fig. 3C), TNFα (Fig. 3D) and VEGF-A (Fig. 3E) were unaffected. The effects of ILK knockdown on signal transduction were examined by Western blotting (Fig. 4A); we found increases in the expression of p-Akt (Fig. 4B), p-GSK-3β (Fig. 4C) and p-P65 (Fig. 4D), but IκBα expression (Fig. 4E) was decreased by fibrin treatment. However, the changes were unaffected by shILK treatment (Fig. 4). Because the inhibition of ILK was dissimilar to the effects of QLT-0267, we further studied the effects of QLT-0267 and shILK treatment on the FAK pathway. p-FAK expression was induced with fibrin and suppressed by QLT-0267 (Fig. 5A), but not by shILK treatment (Fig. 5B). Therefore, QLT-0267 possessed an inhibitory effect on the FAK pathway, which was not related to ILK inhibition.

**Figure 6:**
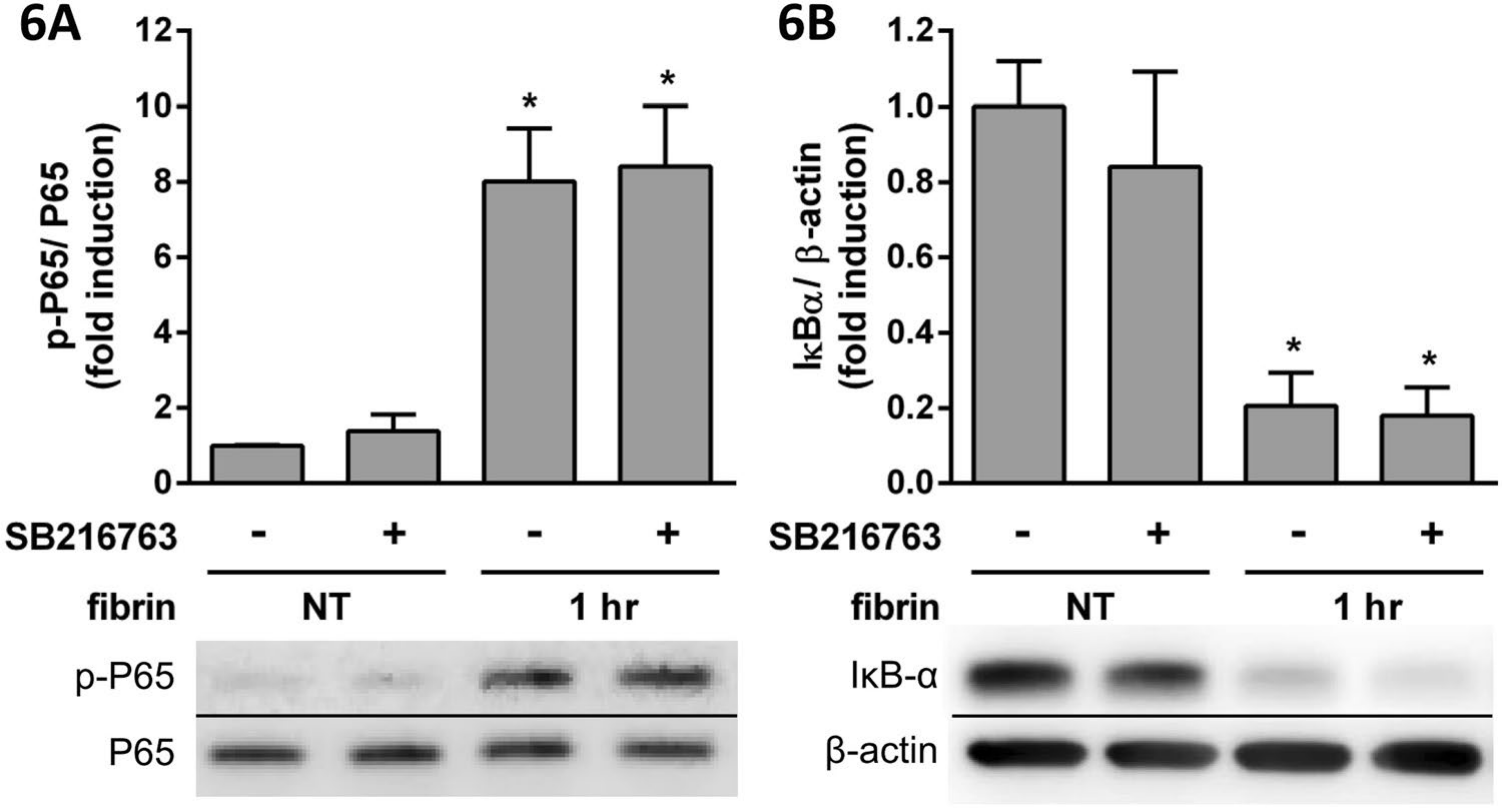
PMCs were overlaid with fibrin via incubation with a fibrinogen and thrombin mixture for 1 hr. The effects of SB216763 treatment on the expression of transduction factors were evaluated by Western blotting. The relative expression of (**A**) p-P65 vs. P65 and (**B**) IκBα vs. β-actin are presented as a percentage of increase over untreated PMCs (NT). *P < 0.05 vs. NT.

**Figure 7:**
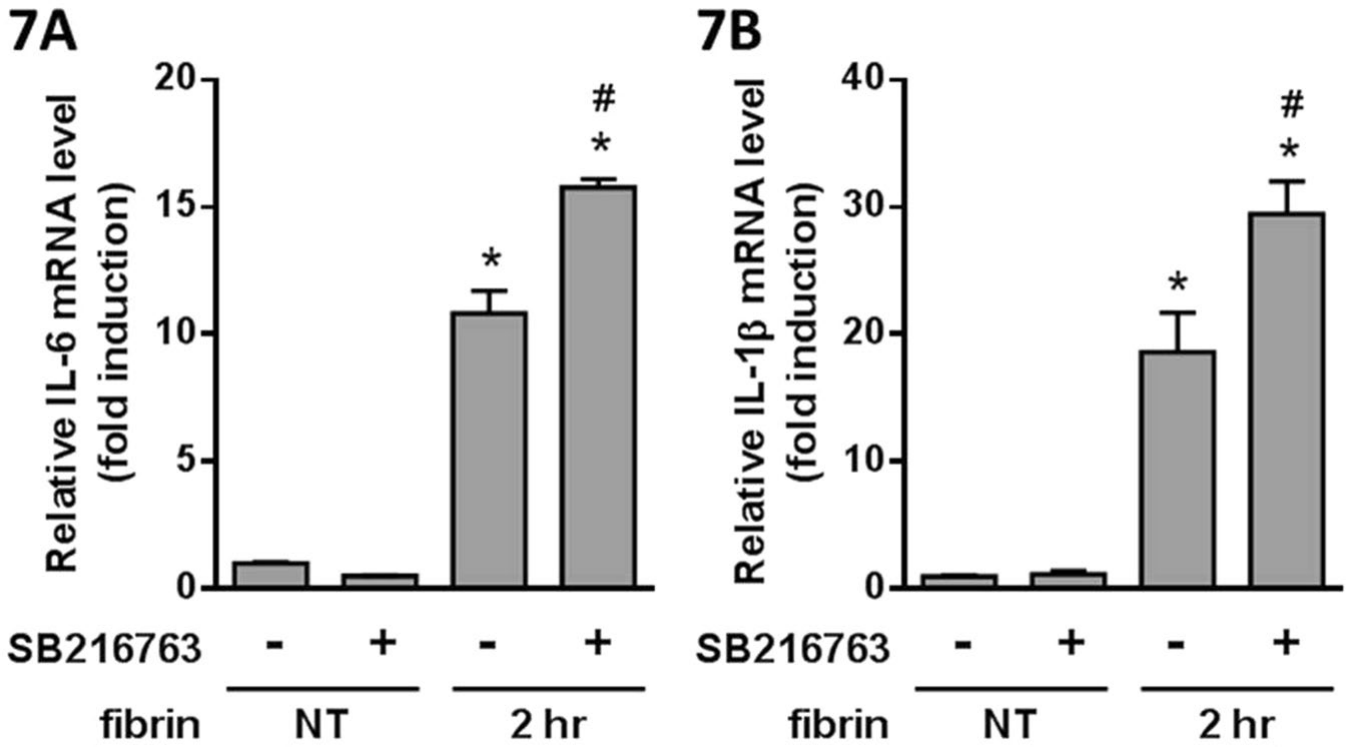
PMCs were overlaid with fibrin via incubation with a fibrinogen and thrombin mixture for 2 hr. The effects of SB216763 treatment on cytokine expression were evaluated by qPCR. The qPCR data for (**A**) IL-6 and (**B**) IL-1β are presented as a percentage of increase over untreated PMCs (NT). *P < 0.05 vs. NT; ^#^*P* < 0.05 vs. fibrin only.

We hypothesized that GSK-3β may be the key pathway involved in the effect of QLT-0267 on cytokine expression. We examined SB216763, a GSK-3β inhibitor^31^, to evaluate its effect on fibrin treatment. SB216763 did not affect the expression of fibrin-induced p-P65 (Fig. 6A) and IκBα (Fig. 6B), whereas fibrin-induced IL-6 (Fig. 7A) and IL-1β (Fig. 7B) expression was augmented.

### The effects of QLT-0267 on an animal model of peritoneal adhesion

**Figure 8:**
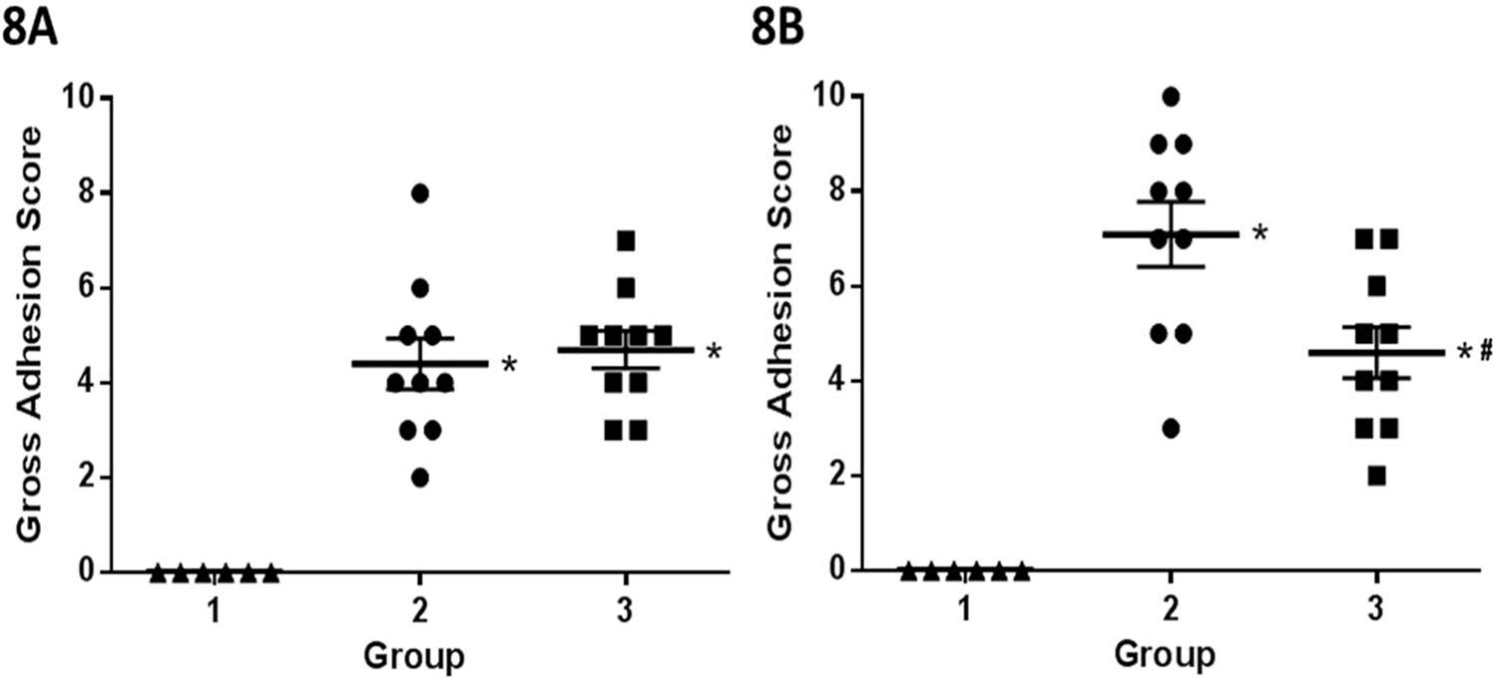
The gross adhesion scores of the postsurgical adhesion mouse model (**A**) one day or (**B**) seven days after the operation. At one day after the operation, the scores from Group 2 (operation, n = 10) and Group 3 (operation with 10 mg/kg QLT-0267 treatment, n = 10) were significantly higher than Group 1 (sham operation, n = 6). At seven days after the operation, the scores from Group 2 were significantly lower than Group 1, while the scores from Group 3 were significantly lower than Group 2. *P < 0.05 vs. Group 1; ^#^*P* < 0.05 vs. Group 2.

**Figure 9:**
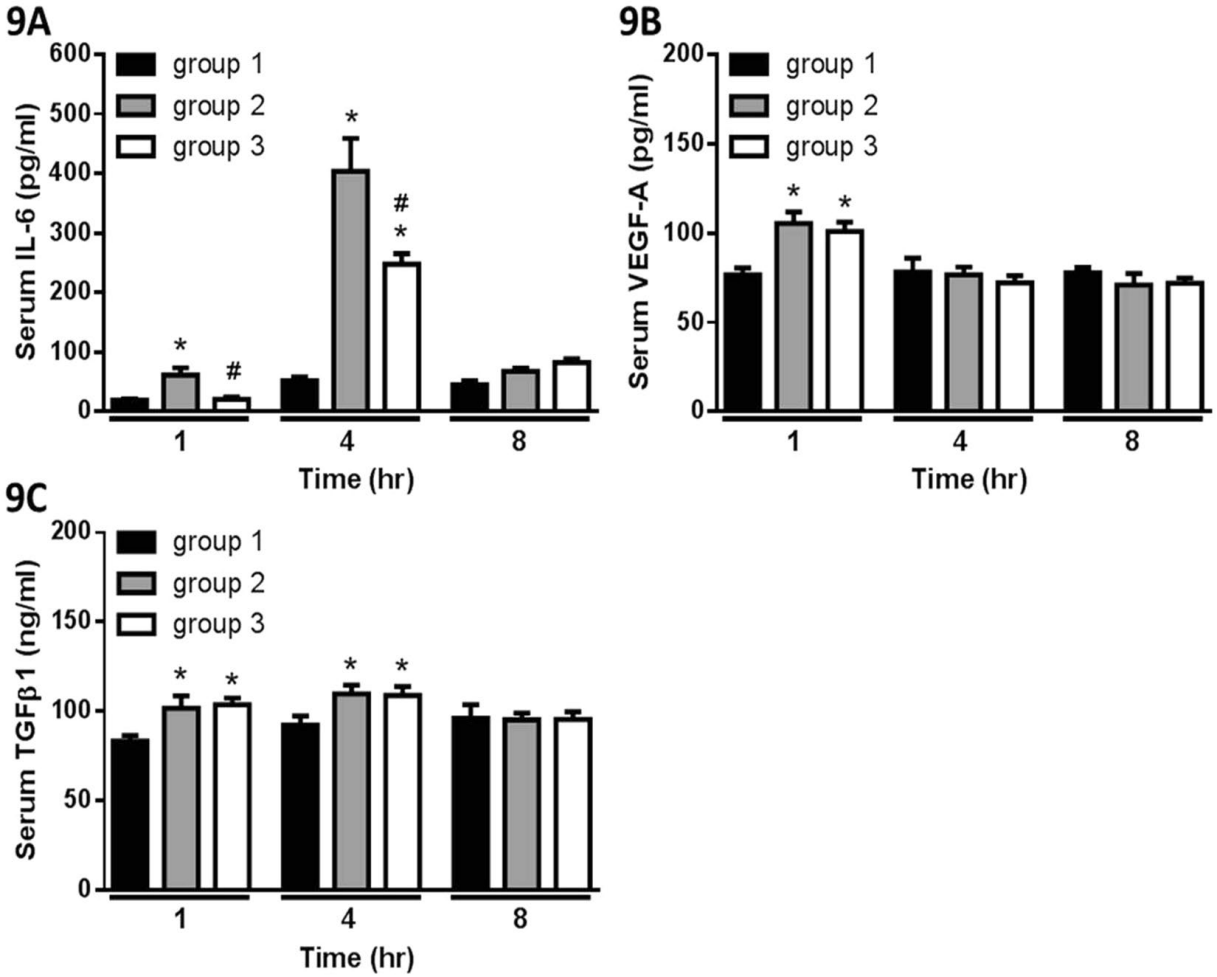
The changes of serum cytokines in the post-surgical adhesion mouse model. The levels of mouse serum (**A**) IL-6, (**B**) VEGF-A and (**C**) TGFβ1 at 1, 4 and 8 hr after the operation are presented. *P < 0.05 vs. Group 1; ^#^*P* < 0.05 vs. Group 2. Group 1, n = 6; Group 2, n = 10; Group 3, n = 10.

A post-surgical adhesion mouse model was used in this study to demonstrate the effect of QLT-0267 in preventing adhesion. One day post-surgery, Group 2 (operation) showed higher adhesion scores than Group 1 (sham operation), but the adhesion scores between Group 2 and Group 3 (operation with QLT-0267 treatment) were similar (Fig. 8A). Seven days post-surgery, Group 2 showed higher adhesion scores than Group 1, and the adhesion score of Group 3 was lower than Group 2 (Fig. 8B). Cytokine changes were evaluated at one, four and eight hours post-surgery. We found elevated levels of mouse serum IL-6 at each time interval post-surgery, and that QLT-0267 treatment suppressed IL-6 elevation at one and four hours after the operation (Fig. 9A). QLT-0267 treatment did not suppress the elevation of VEGF-A (Fig. 9B) and TGFβ1 (Fig. 9C), and the levels of mouse serum IL-1β and TNFα were too low to be detected by ELISA.

## Discussion

In this study, we demonstrated that fibrin induced IL-1β, IL-6, TNFα and VEGF-A expression in PMCs, which could be attenuated by QLT-0267, and that QLT-0267 treatment could ameliorate post-surgical peritoneal adhesion. These findings showed that fibrin not only apposes two peritoneal surfaces, but could also augment peritoneal inflammation and cause peritoneal adhesions. Furthermore, we illustrated that ILK inhibition is not the sole regulator of the mechanism of QLT-0267. We also performed *in vivo* studies to examine the effects of QLT-0267 in preventing post-surgical peritoneal adhesion. Our mouse model study showed that QLT-0267 could decrease the severity of peritoneal adhesion and reduce serum IL-6 levels. These data support the use of QLT-0267 as a new strategy in preventing peritoneal adhesion.

Several studies have shown that fibrin may promote inflammatory reactions, and is related to many diseases, such as arthritis^32^, Alzheimer’s disease^33^, renal interstitial fibrosis^34^ and dystrophic muscle fibrosis^35^. At cellular levels, several studies have shown that fibrin and/or fibrinogen effect cytokine modulation. Pancreatic stellate cells have been shown to interact with fibrin and fibrinogen to induce many cytokines, including IL-6, IL-8, monocyte chemoattractant protein-1 (MCP-1) and VEGF^36^. Fibrinogen induces IL-1β production from mouse macrophages, and thereby drives TGFβ synthesis^35^. A study investigating a co-culture of myeloma cell lines and bone marrow stromal cells found that QLT-0267 decreased VEGF and IL-6 secretion in the latter^25^. Similarly, investigations on diabetic retinopathy and glioblastoma cell lines found that QLT-0267 reduced VEGF expression from diabetic retinal tissue^37^ and the glioblastoma cells^38^, respectively. Therefore, our study and these investigations provided evidence to support the therapeutic role of QLT-0267 in many diseases relative to inflammatory cytokines.

The mechanism of the effect of QLT-0267 has been shown to involve ILK inhibition^26^; however, we found that ILK knockdown via treatment of PMCs with shILK only affected IL-6 expression. Therefore, there are likely other mechanisms by which QLT-0267 affects other fibrin-induced cytokines. We found that the FAK pathway was involved in fibrin-induced cytokine expression and was affected by QLT-0267, which has previously been proven in squamous cell carcinoma cells^39^. Through our experiments on signalling pathways, we found that the effects of QLT-0267 were mediated by the GSK-3β pathway without involving P65 phosphorylation and IκBα degradation. The observation that GSK-3β regulates NF-κB function without affecting phosphorylation of P65 and IκBα degradation has been reported in a previous study on pancreatic cancer cells^40^.

**Figure 10:**
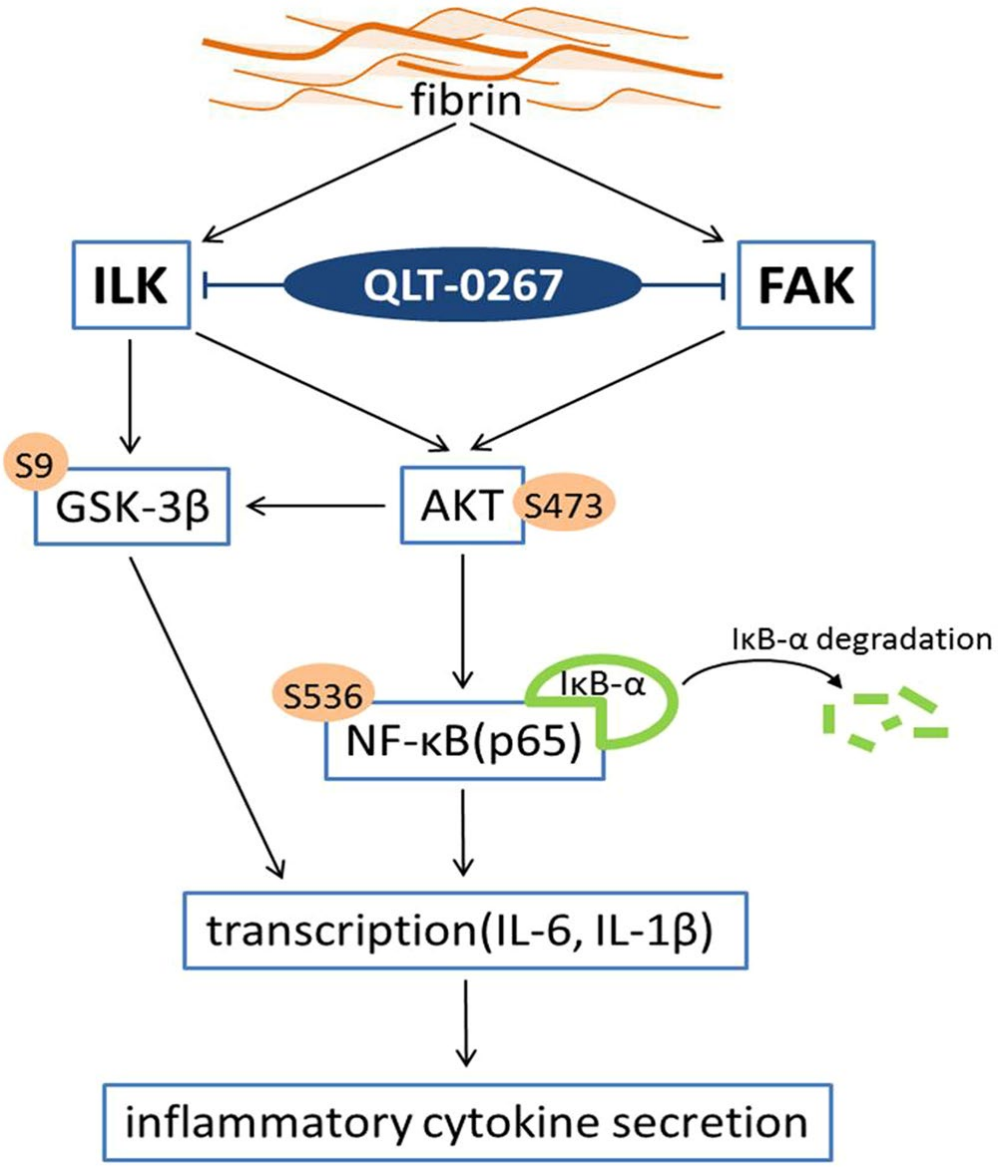
Diagram showing fibrin and QLT-0267 involvement in the pathogenesis of peritoneal adhesion. Peritoneal insults induce the release of inflammatory cytokines, which promotes fibrin formation. Fibrin deposition on PMCs induces cytokine production from the PMCs, which further promotes peritoneal adhesion. QLT-0267 has inhibitory effects on both the ILK and FAK pathways.

Although fibrin has been implicated in the pathogenesis of peritoneal adhesion^11,14^, it has not been shown to induce inflammatory cytokines. Our study indicates the existence of another mechanism by which fibrin promotes peritoneal adhesion (Fig. 10). Peritoneal insults induce the release of inflammatory cytokines, which promotes fibrin formation. Fibrin deposition on the PMCs induces cytokine production from the PMCs, which further promotes peritoneal adhesion. QLT-0267 could block the production of fibrin-induced cytokines and thereby prevent peritoneal adhesion. A variety of barriers are available in clinical use, but an optimal material has not yet been determined. Furthermore, the optimization or functionalization of barrier materials that inhibit the pathogenesis of peritoneal adhesion could be an innovative future strategy in preventing peritoneal adhesion^17^. Using QLT-0267 intraperitoneally in our animal model provided a clue to combining the use of barriers with QLT-0267 to achieve better prevention of peritoneal adhesion^41^.

This study was limited because our animal model did not directly demonstrate the effects of fibrin on cytokines, although our *in vitro* study showed the fibrin could induce cytokine expression from mesothelial cells. We designed the animal model to be clinically relevant instead of only being fibrin-related. Nevertheless, we successfully demonstrated the suppressive effects of QLT-0267 in our animal study, which might indicate that the effects of fibrin were inhibited via the ILK- and FAK-related pathways. Our previous study demonstrated fibrin could induce epithelial-to-mesenchymal transition (EMT) on human PMCs^42^. In the present study, we performed Western blots to evaluate cell marker changes after fibrin treatment in rat PMCs. However, the changes of the cell markers were not obvious after fibrin treatment in rat PMCs (Supplementary Figure 5S). We assume the EMT phenomenon is not easy to induce on rat PMCs by fibrin.

In conclusion, our study provided novel evidence that fibrin-induced cytokine production may involve in the mechanism of peritoneal adhesion formation. Furthermore, treatment with QLT-0267 is a new strategy in preventing peritoneal adhesion in patients undergoing abdominal surgery.

## Electronic supplementary material

Supplementary Information
