# Supplementary material for: The Small Molecule Inhibitor QLT-0267 Decreases the Production of Fibrin-Induced Inflammatory Cytokines and Prevents Post-Surgical Peritoneal Adhesions
Source: Sci Rep. 2018 Jun 21;8:9481. doi: 10.1038/s41598-018-25994-5 (PMC6013455; doi:10.1038/s41598-018-25994-5)

The Small Molecule Inhibitor QLT-0267 Decreases the Production of Fibrin-Induced Inflammatory Cytokines and Prevents Post-Surgical Peritoneal Adhesions

Cheng-Chung Fang, Tzung-Hsin Chou, Jenq-Wen Huang, Chien-Chang Lee, Shyr-Chyr Chen

Supplementary Table S1. RT-PCR primers

Primers		Sequences	product (bp)
IL-6	sense	5'-GAGGATACCACCCACACCAGACCAGTA-3'	525
	antisense	5'-GGTTTGCCGAGTAGACCTCATAGTGAC-3'	
IL-1β	sense	5'-GGGATGATGACGACCTGCTA-3'	192
	antisense	5'-TGTCGTTGCTTGTCTCTCCT-3'	
TNFα	sense	5'-GAAACACACGAGACGCTGAA-3'	400
	antisense	5'-CCTGAAGACAGCTTCCCAAC-3'	
VEGF-A	sense	5'-AGACCTCTCACCGGAAAGAC-3'	445
	antisense	5'-ACTCAAGTCCACAGCAGTCA-3'	
TGFβ1	sense	5'-CCTGCAAGACCATCGACATG-3'	337
	antisense	5'-ACTGCTTCCCGAATGTCTGA-3'	
VEGF-C	sense	5'-CTGTGGACCCAACAAGGAGT-3'	243
	antisense	5'-CAGGCACATTTTCCAGGATT-3'	
GAPDH	sense	5'-AGACAGCCGCATCTTCTTGT-3'	239
	antisense	5'-TTCCCATTCTCAGCCTTGAC-3'	

Supplementary Table S2. Quantitative Real-Time PCR primers

Primers		Sequences
IL-6	sense	5'-CCAGCCAGTTGCCTTCTT-3'
	antisense	5'-AATTAAGCCTCCGACTTGTGAA-3'
IL-1β	sense	5'-TCATTGTGGCTGTGGAGAAG-3'
	antisense	5'-CACTAGCAGGTCGTCATCATC-3'
TNFα	sense	5'-CGTGTTCATCCGTTCTCTACC-3'
	antisense	5'-GCCACTACTTCAGCGTCTC-3'
VEGF-A	sense	5'-GCGGATCAAACCTCACCAAA-3'
	antisense	5'-CTTGGCTTGTCACATTTTTC-3'
ILK	sense	5'-ACAGACGCTCAGCAGACA-3'
	antisense	5'-GCCACCTTCATTCCAATCTCC-3'
18S	sense	5'-GGAGAGGGAGCCTGAGAAAC-3'
	antisense	5'-CCTCCAATGGATCCTCGTTA-3'

Supplementary Table S3. Gross adhesion scoring system

Strength	Score	Total area (cm^2)	Score
No adhesions	0	No adhesions	0
Thin	1	< 0.5	1
One definite localized adhesions	2	0.5 ~ 0.99	2
Two definite localized adhesions	3	1 ~ 1.49	3
Three definite localized adhesions	4	1.5 ~ 1.99	4
> four definite localized adhesions	5	> 2	5

Supplementary Figure 1S. The immunofluorescence stains of rat peritoneal mesothelial cells (PMCs). The cell markers of PMCs were simultaneously stained by (A) cytokeratin-18 antibody and (B) α-smooth muscle actin antibody, and (C) the nuclei were stained by 4',6-diamidino-2-phenylindole (DAPI).

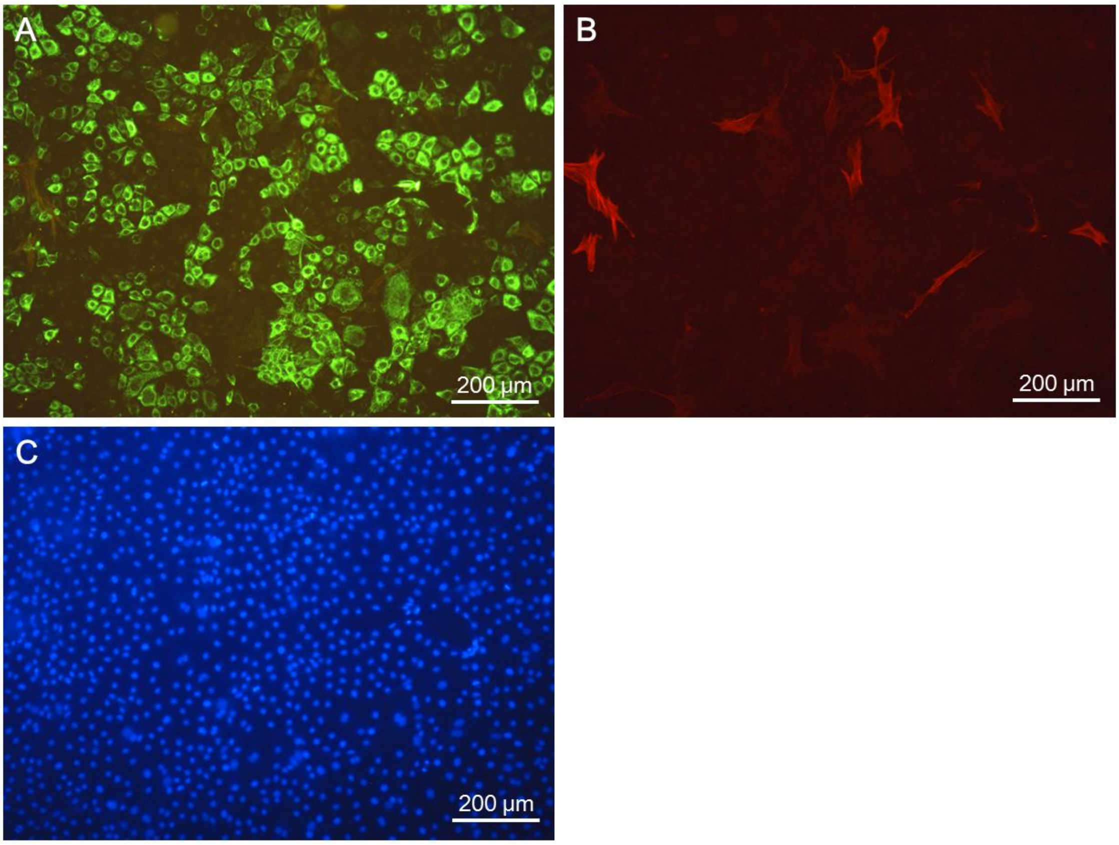

Supplementary Figure 2S. The histology of mice tissue stained by haematoxylin and eosin. The mice tissue sections were displayed in (A) sham operation, (B) 1 hour after operation, (C) 4 hours after operation, (D) 1 day after operation, and (E) 7 days after operation. There were fibrin depositions on the parietal peritoneum after gauze injury at 1 and 2 hours post operation. The adhesion between abdominal wall and intestinal wall consisted of fibrin and leukocytes one day after operation. The adhesion between abdominal wall and intestinal wall consisted of collagen fibers, fibroblasts, capillaries and mononuclear cells 7 days after operation.

SK: skeletal muscle; SM: smooth muscle; Arrow: adhesion between abdominal wall and intestinal wall.

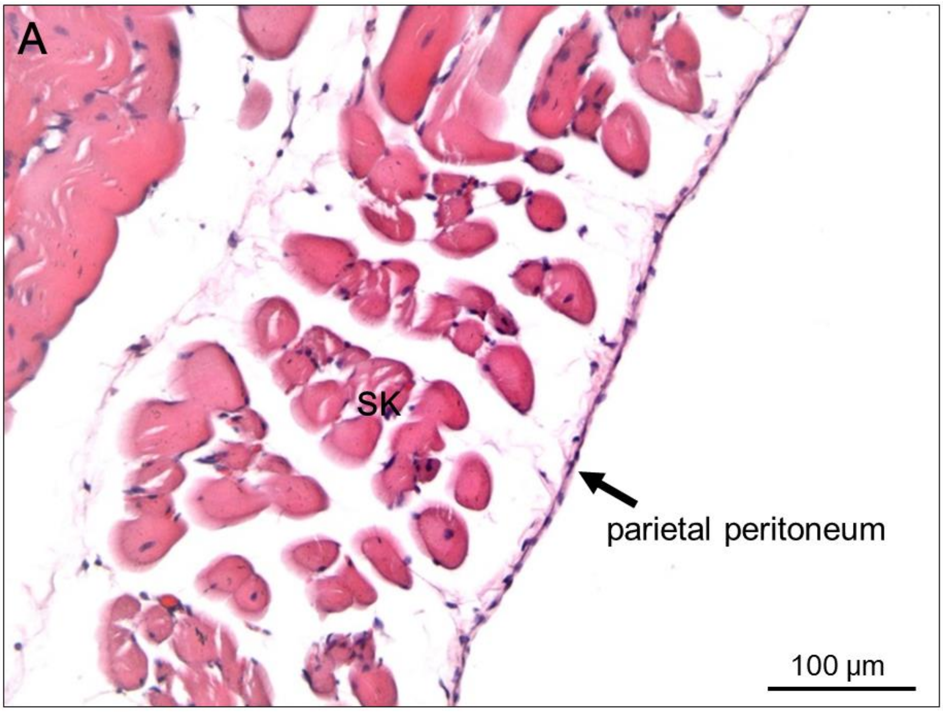

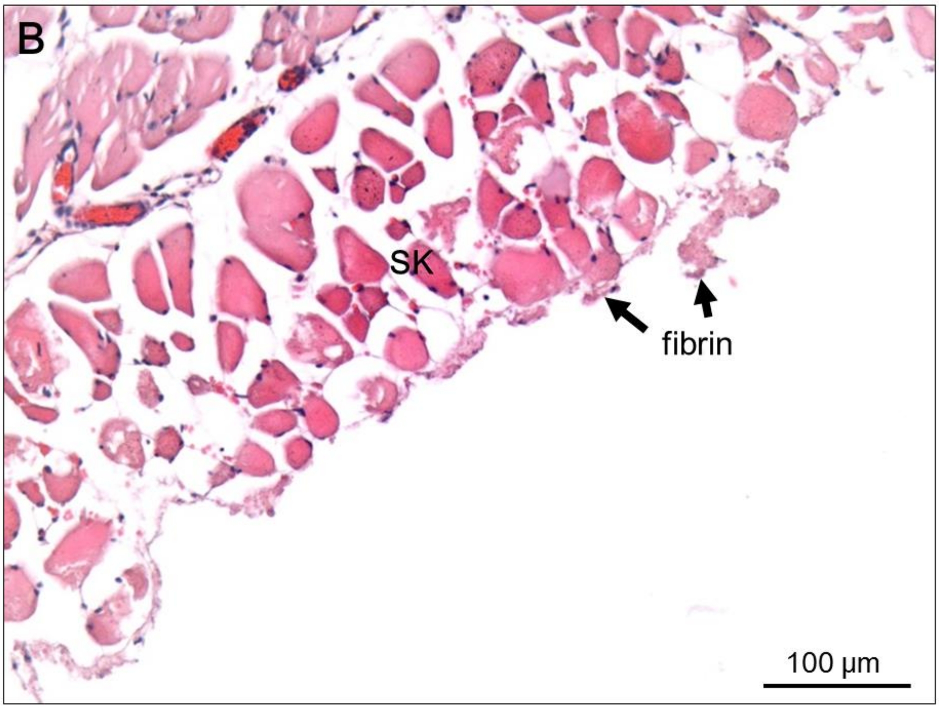
B
SK
fibrin
100 µm

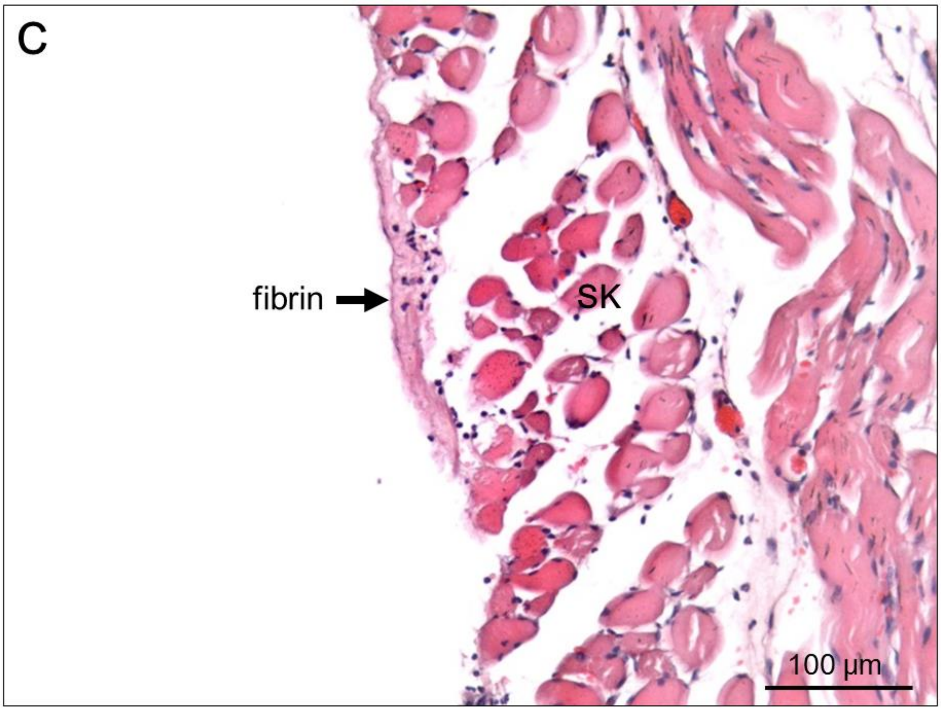
C
fibrin
SK
100 µm

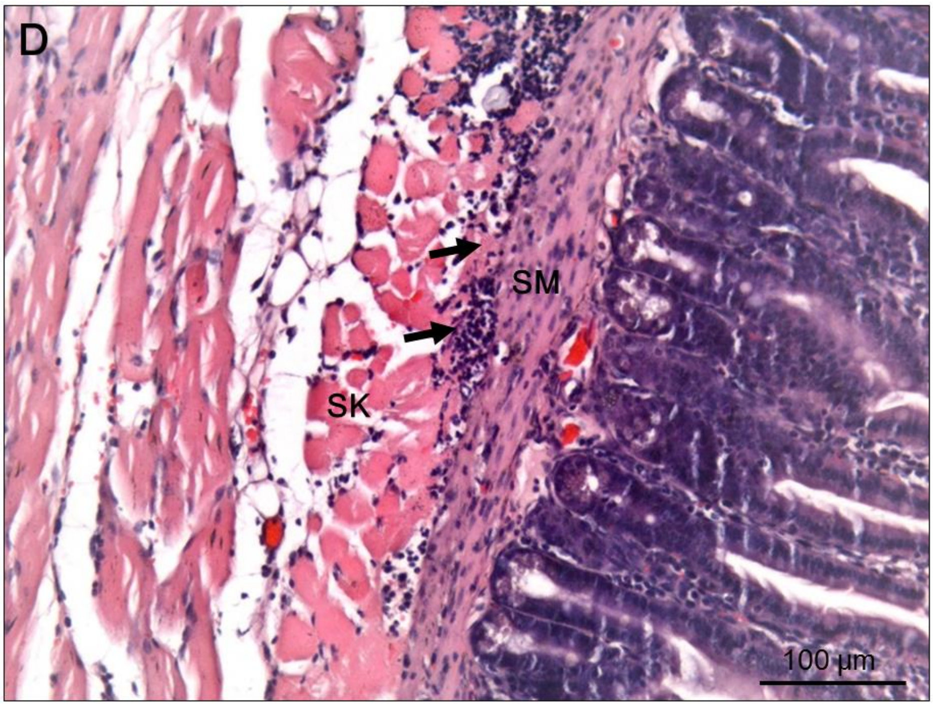
D
SM
SK
100 µm

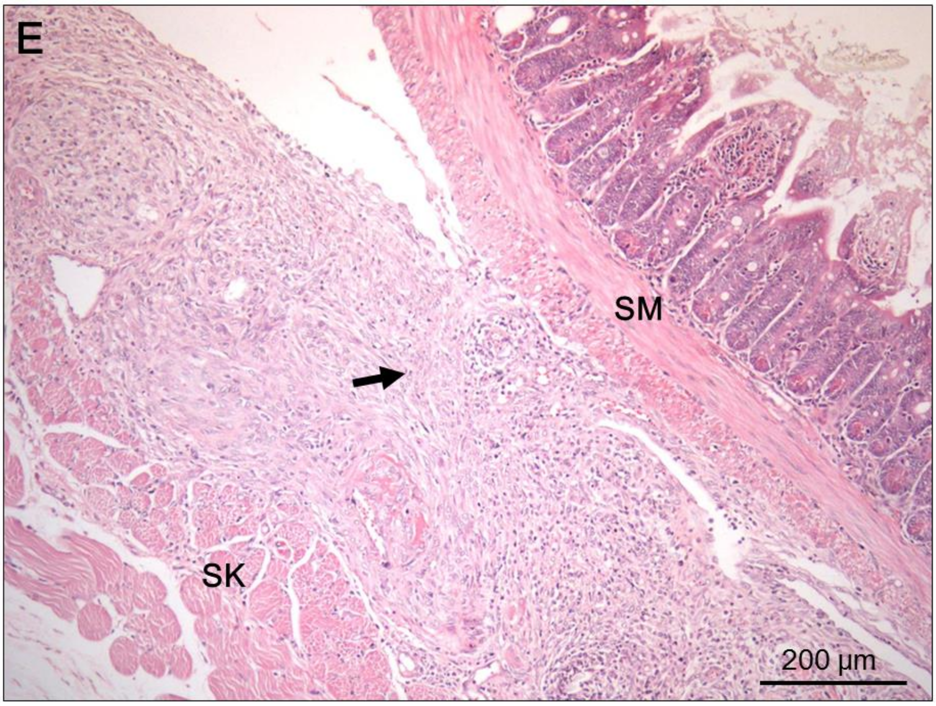
E
SM
SK
200 µm

Supplementary Figure 3S. Examples of gross appearance in abdominal cavity 7 days after operation. There were no adhesions in Group 1 mice (sham operation, A). The adhesions (arrow) were formed after parietal peritoneum were injured by gauze in Group 2 mice (operation, B) and Group 3 mice (operation with 10 mg/kg QLT-0267 treatment, C).

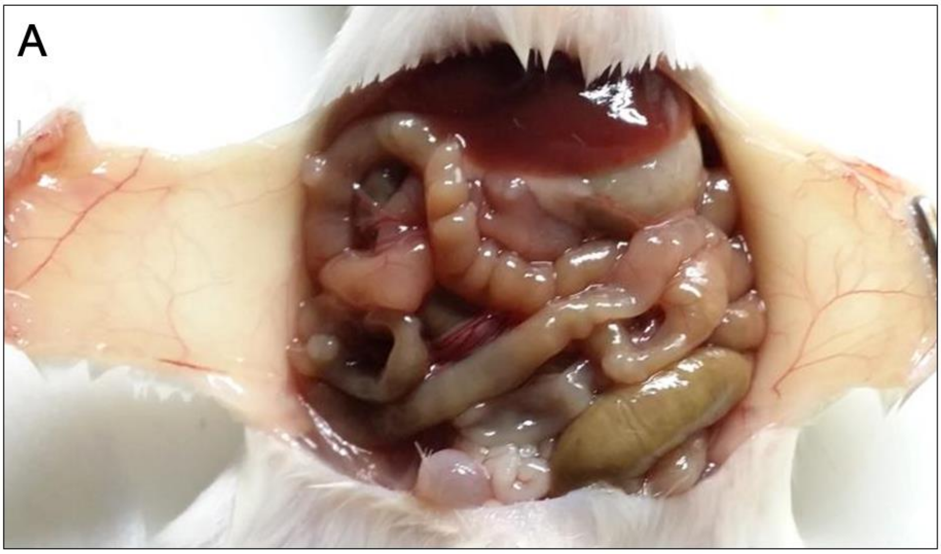

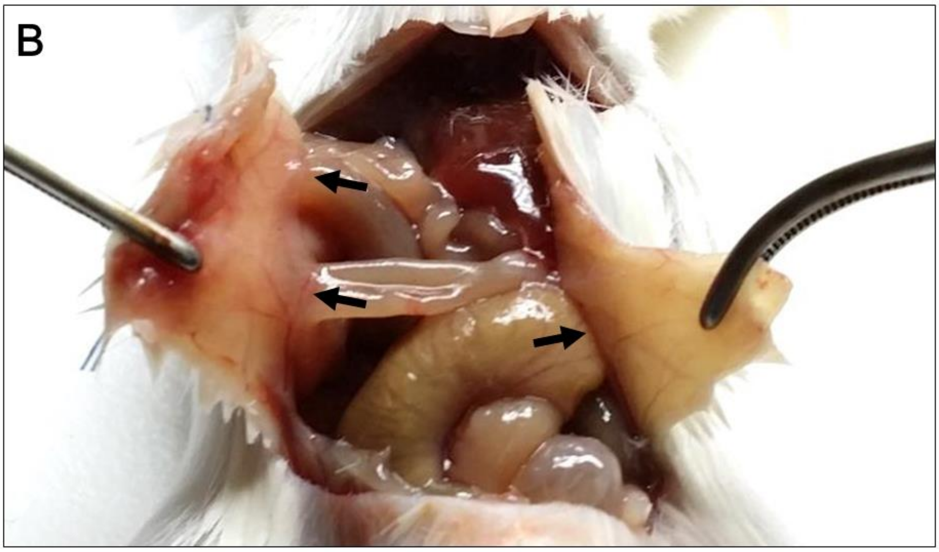

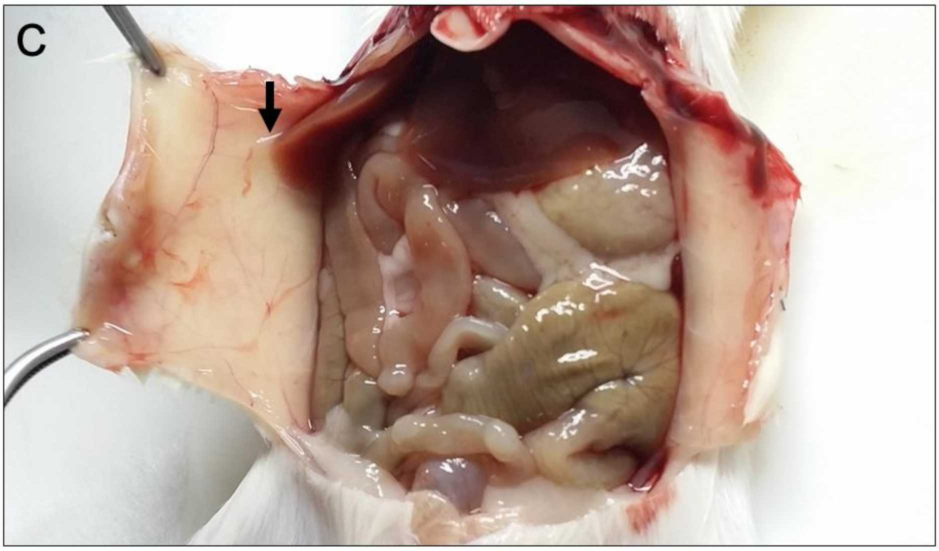
C

Supplementary Figure 4S. Original full-length gel images of RT-PCR. Lane 2 was treated with 10 mg/ml of bovine serum albumin (BSA) to be a negative control to fibrin. However, we found that BSA was not a negative control due to contamination by fibrinogen. Therefore, we removed the BSA lanes in Figure 1. The data of 6-hr results were omitted in Figure 1 for easy reading. Lane 1: untreated; lane 2: BSA; lane 3: fibrin; lane 4: fibrin + 2 μM QLT-0267; lane 5: fibrin + 5 μM QLT-0267; lane 6: fibrin + 10 μM QLT-0267.

Fig. 1A - IL-6

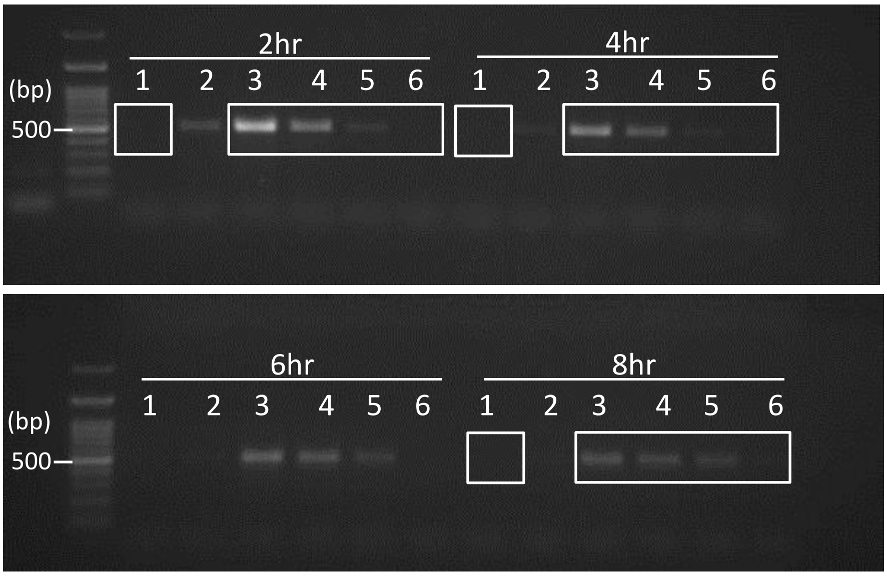

Fig. 1A - IL-1

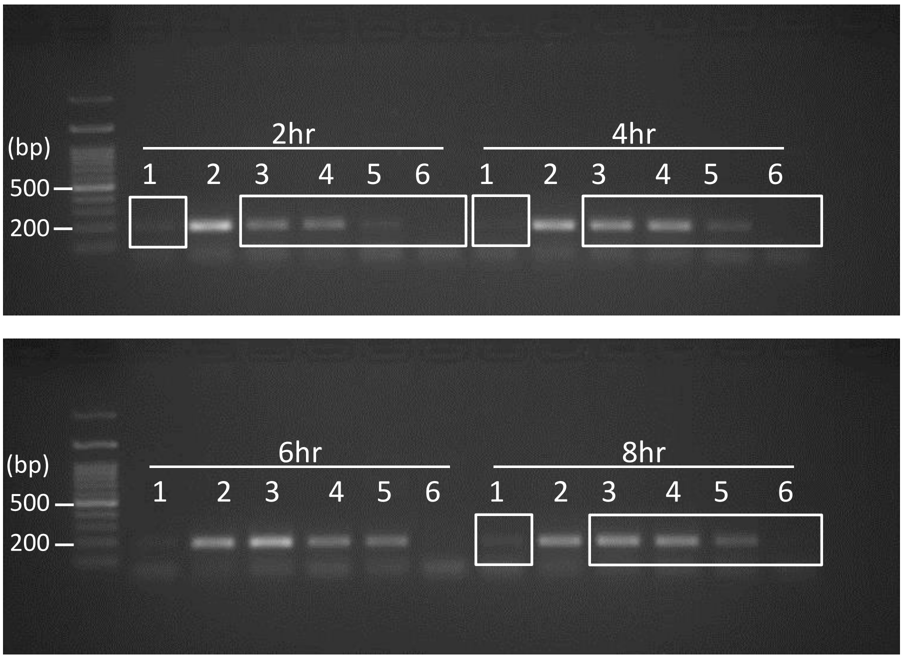

Fig. 1A - TNFα

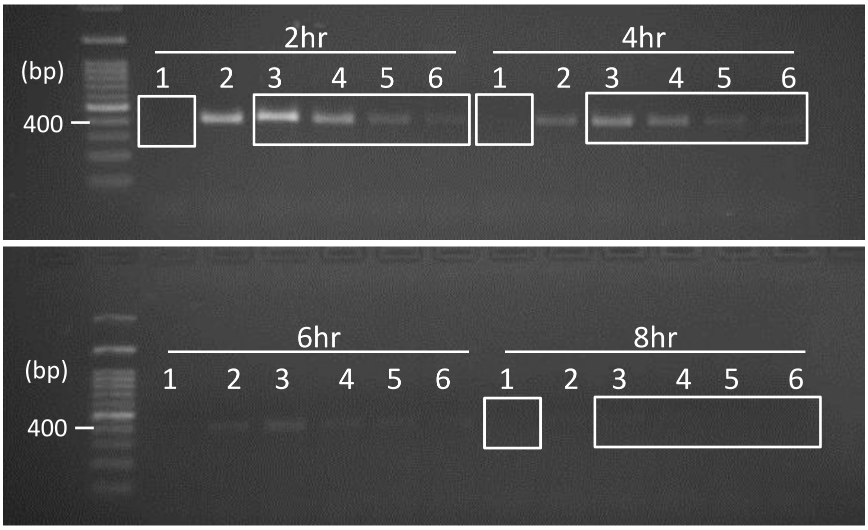

Fig. 1A - VEGF-A

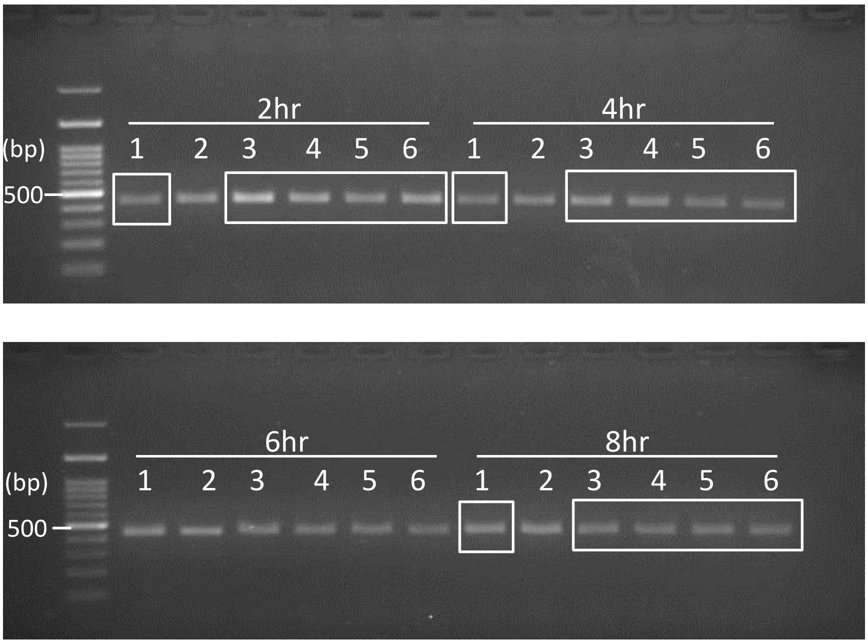

Fig. 1A - TGFβ1

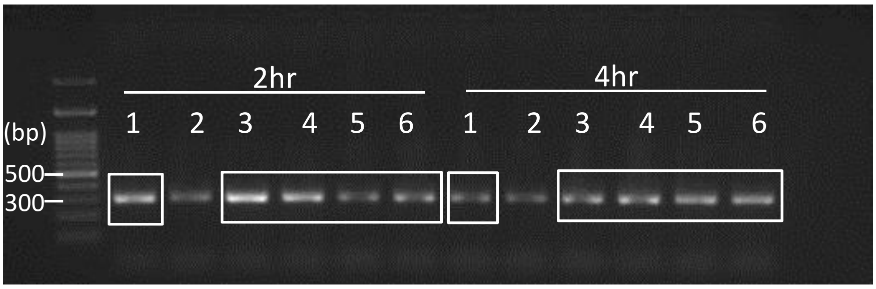

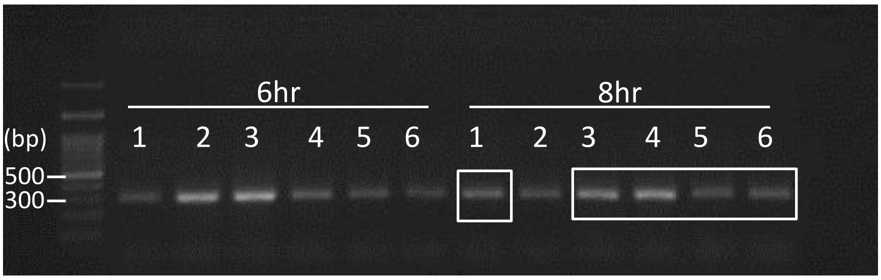

Fig. 1A - VEGF-C

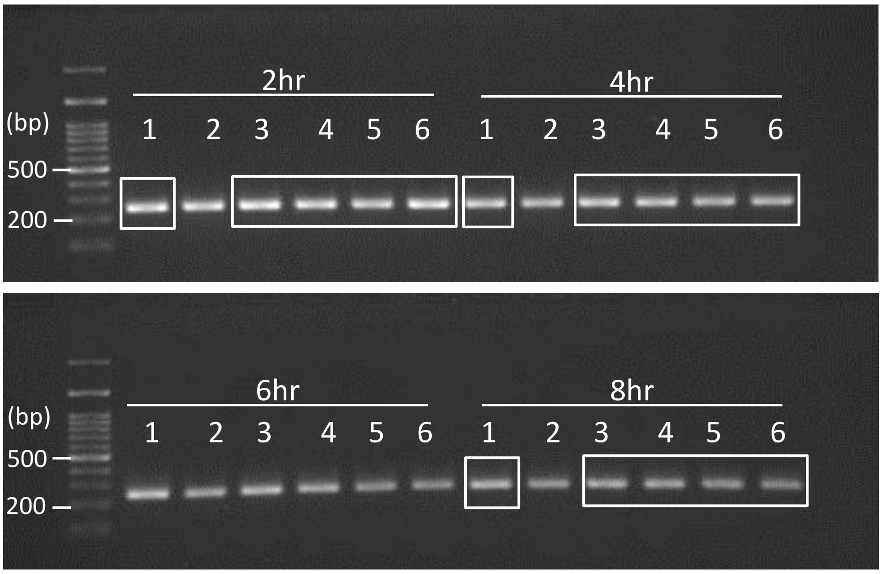

Fig. 1A - GAPDH

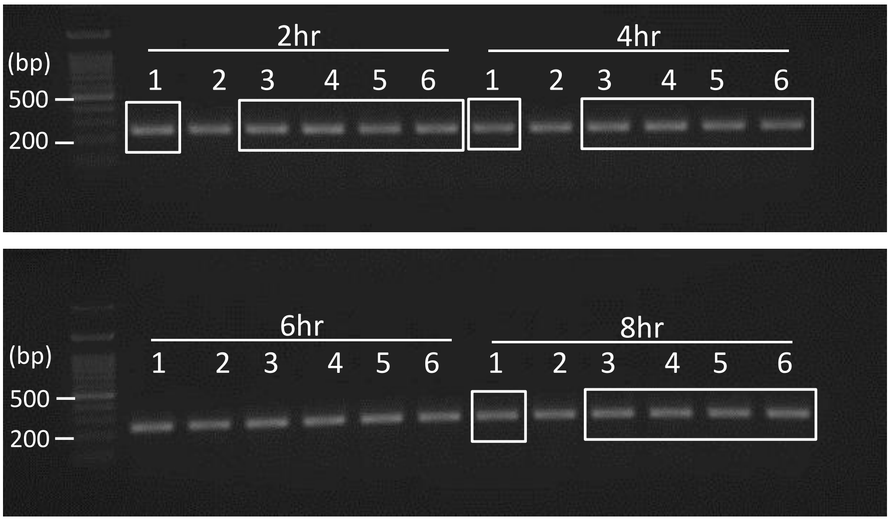

Supplementary Figure 5S. Western blots of cell markers in fibrin-covered rat peritoneal mesothelial cells (PMCs). PMCs were overlaid with fibrin via incubation with a fibrinogen and thrombin mixture for 0, 1, 4 and 8 hr. The effects of fibrin with/without QLT-0267 treatment on the expression of cell markers were examined by Western blotting. The fibronectin, αv integrin, snail and α-smooth muscle actin (α-SMA) did not increase significantly after fibrin treatment, and cytokeratin-18 (CK-18) did not decrease significantly after fibrin treatment.

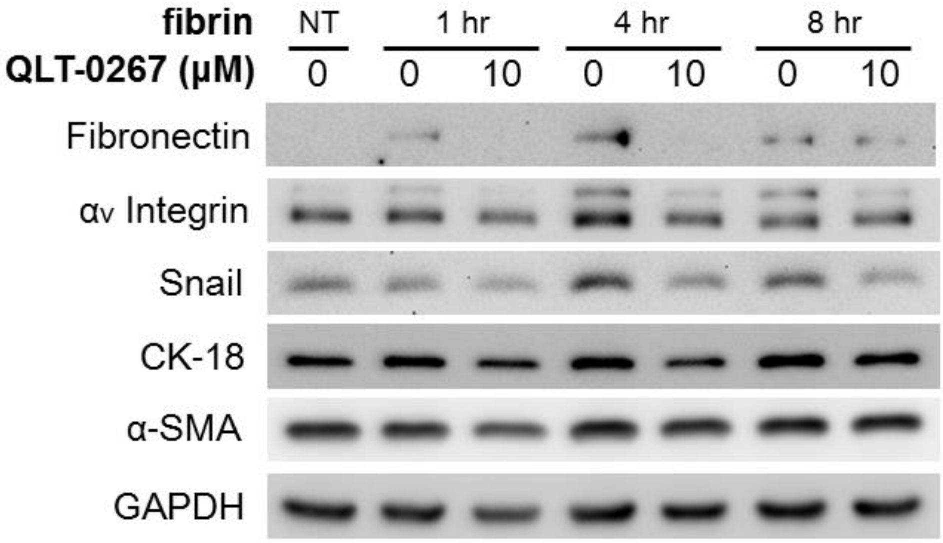

Supplementary Figure 6S. Unprocessed original images of western blots.

Groups of figure 2:

Lane 1: untreated; lane 2: 1-hr fibrin; lane 3: 1-hr fibrin + 10 μM QLT-0267; lane 4: 2-hr fibrin; lane 5: 2-hr fibrin + 10 μM QLT-0267.

Related to fig. 2A

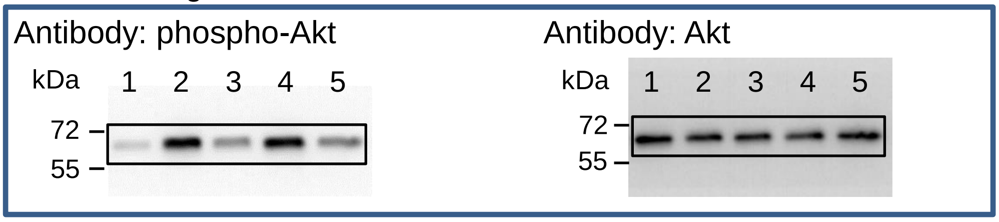

Related to fig. 2B

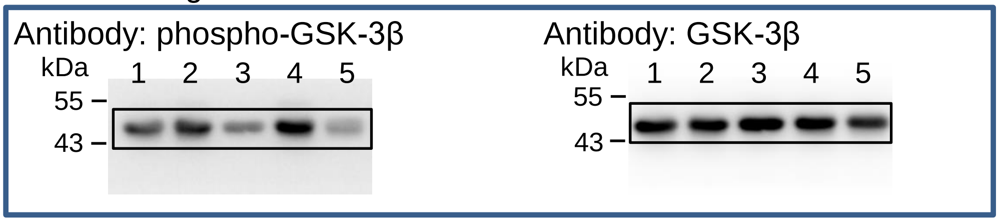

Related to fig. 2C

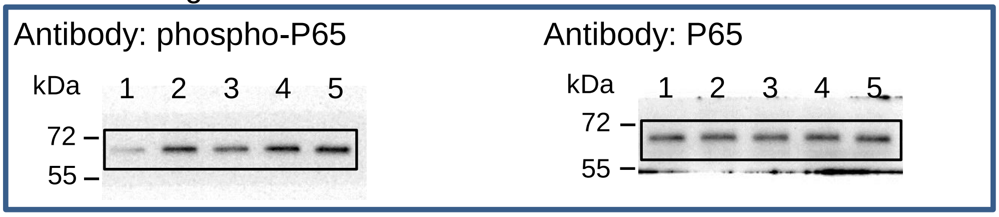

Related to fig. 2D

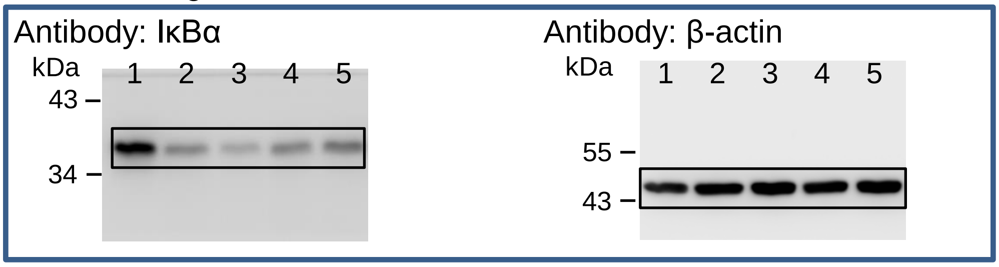

Groups of figure 4:

Lane 1: empty vector (EV) control; lane 2: shILK; lane 3: EV + 1-hr fibrin; lane 4: shILK + 1-hr fibrin; lane 5: EV + 2-hr fibrin; lane 6: shILK + 2-hr fibrin.

Related to fig. 4A

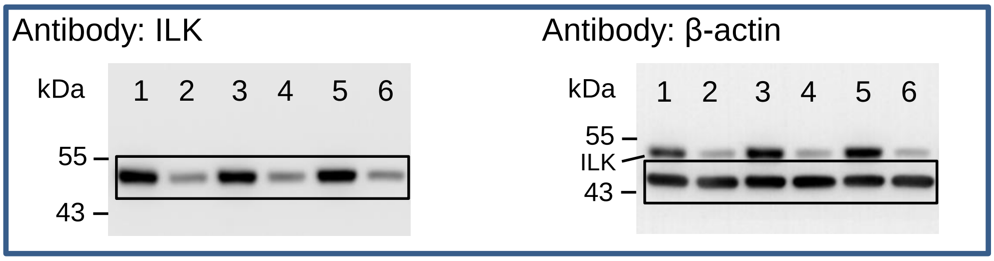

Related to fig. 4B

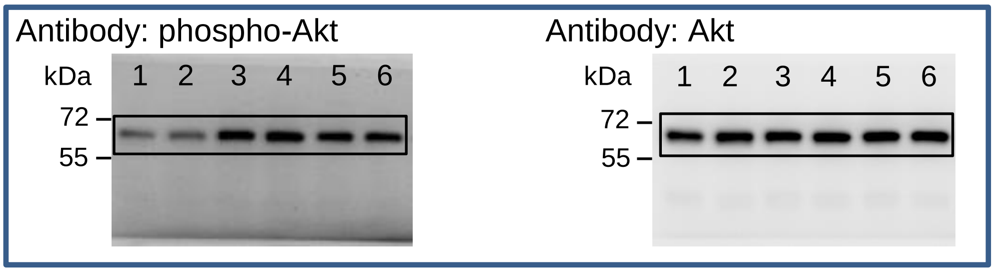

Related to fig. 4C

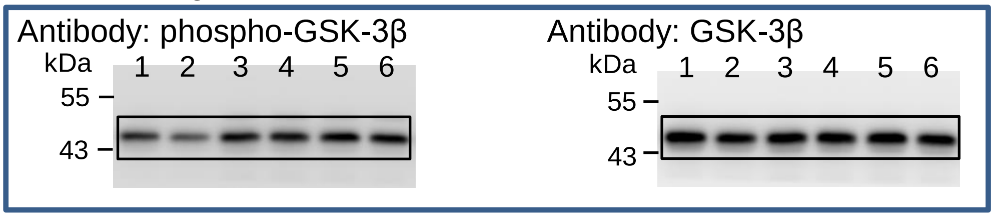

Related to fig. 4D

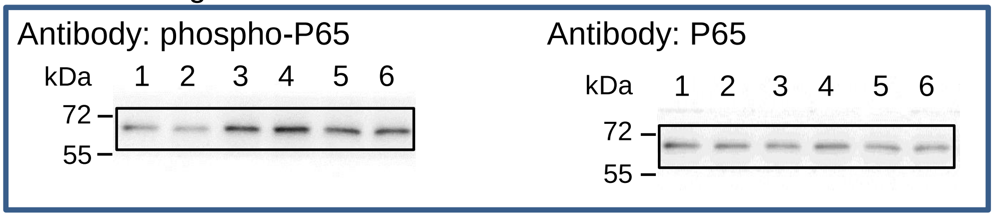

Related to fig. 4E

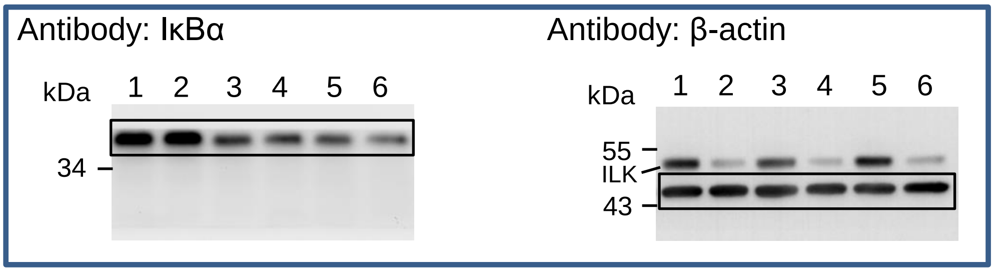

Groups of figure 5A:

Lane 1: untreated; lane 2: 1-hr fibrin; lane 3: 1-hr fibrin + 10 μM QLT-0267; lane 4: 2-hr fibrin; lane 5: 2-hr fibrin + 10 μM QLT-0267.

Related to fig. 5A

Antibody: phospho-FAK

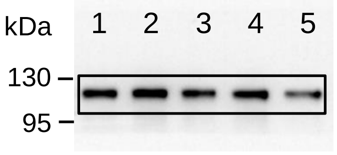

Antibody: FAK

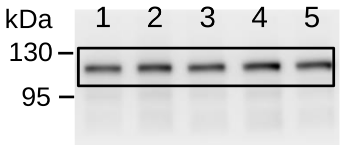

Groups of figure 5B:

Lane 1: empty vector (EV) control; lane 2: shILK; lane 3: EV + 1-hr fibrin; lane 4: shILK + 1-hr fibrin; lane 5: EV + 2-hr fibrin; lane 6: shILK + 2-hr fibrin.

Related to fig. 5B

Antibody: phospho-FAK

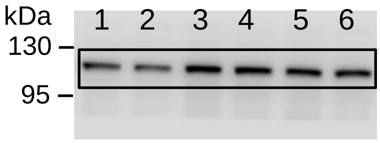

Antibody: FAK

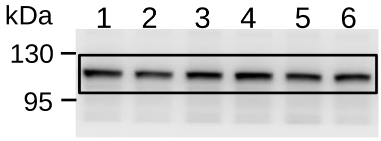

Groups of figure 6:

Lane 1: untreated; lane 2: 2 μM SB216763; lane 3: 1-hr fibrin; lane 4: 1-hr fibrin + 2 μM SB216763.

Related to fig. 6A

Antibody: phospho-P65

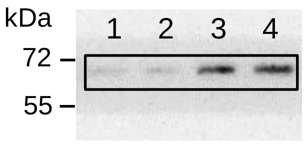

Antibody: P65

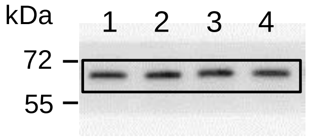

Related to fig. 6B

Antibody: IκBα

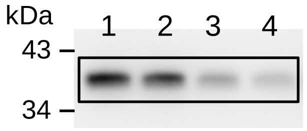

Antibody: β-actin

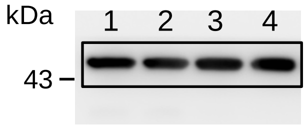

Groups of supplementary figure 5S:

Lane 1: untreated; lane 2: 1-hr fibrin; lane 3: 1-hr fibrin + 10 μM QLT-0267; lane 4: 4-hr fibrin; lane 5: 4-hr fibrin + 10 μM QLT-0267; lane 6: 8-hr fibrin; lane 7: 8-hr fibrin + 10 μM QLT-0267.

Related to supplementary fig. 5S

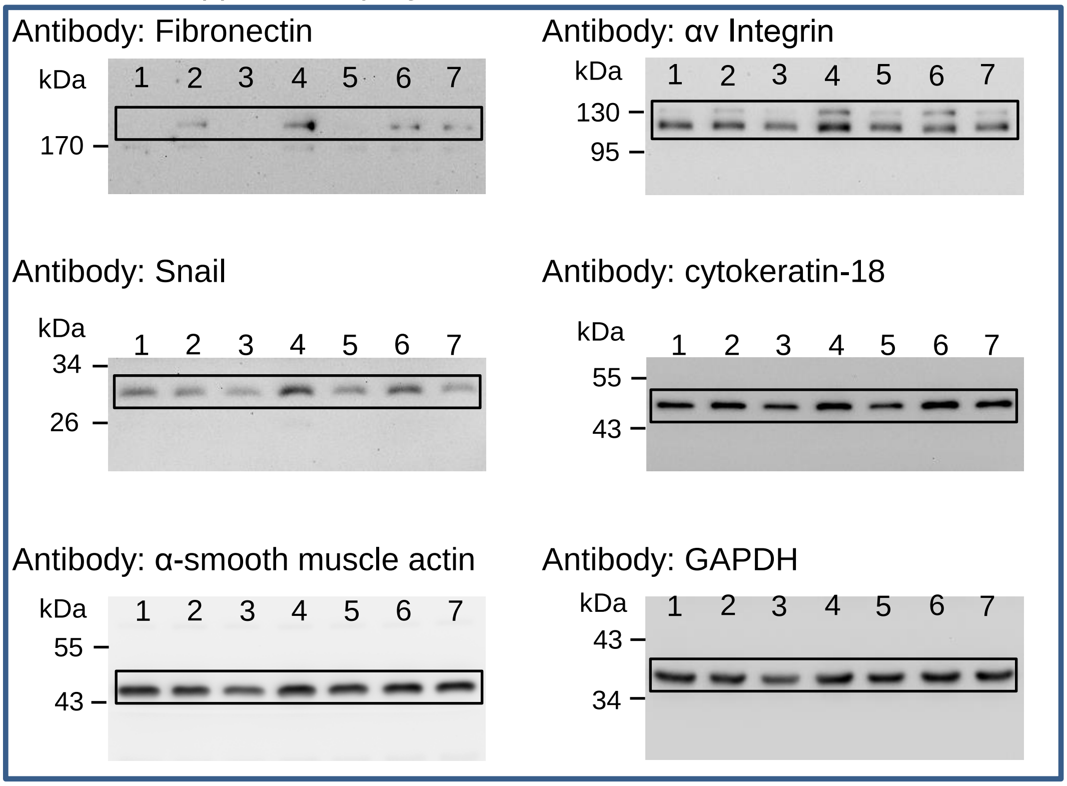

Supplement: Supplementary file 1 — Supplementary Information [file 41598_2018_25994_MOESM1_ESM.pdf]
